# Block CO_2_–Polycarbonates: Tunable Chain Extension with Zn(II) Carboxylates

**DOI:** 10.1021/acsmacrolett.5c00639

**Published:** 2025-11-05

**Authors:** Kam C. Poon, Chang Gao, Diego A. Resendiz-Lara, Mantas Drelingas, Charlotte K. Williams

**Affiliations:** † Chemistry Research Laboratory, Department of Chemistry, 6396 University of Oxford, Oxford OX1 3TA, U.K.

## Abstract

Low molecular weight α,ω-dicarboxylic acid triblock polymers, featuring polycarbonate blocks flanking a central polyester block, are synthesized and assembled into dynamic halatopolymers via Zn­(II) coordination. The number of chains coupled through Zn­(II)–carboxylate interactions is tuned by using 4-*tert*-butylbenzoic acid (tBBA) as a sterically hindered capping ligand. Reducing tBBA content vs polymer and zinc content increases the number of junctions connected and the effective halatopolymer molar mass, leading to polymers with higher zero-shear viscosities, slower relaxation dynamics, and more pronounced elastic behavior. Temperature ramp, time–temperature superposition, and creep recovery experiments confirm enhanced dimensional stability and reduced flow under load as the degree of chain coupling increases. In the absence of Zn­(II), the polymers display purely viscous behavior, highlighting the critical role of reversible metal–ligand interactions in network formation. This strategy provides a modular route to convert synthetically accessible, renewably sourced, low molar mass polymers into tunable, functional materials, offering a design platform for a new generation of polymeric materials.

## Graphical abstract

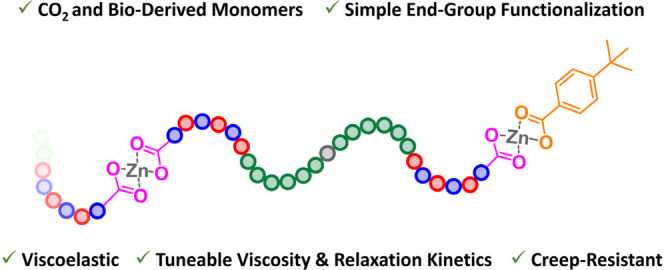

Designing the next generation of polymeric materials demands a shift toward sustainability without compromising functionality.^1–4^ As pressure mounts to reduce dependence on fossil resources and improve end-of-life outcomes, new strategies are needed to create high-performance polymers from renewable or waste-derived feedstocks.^5–7^ This challenge calls for precision chemistries to transform simple building blocks into tunable, functional materials.^8–10^

Heterocycle ring-opening polymerization (ROP) and heterocycle/heteroallene ring-opening copolymerization (ROCOP) are powerful techniques for synthesizing aliphatic polyesters and polycarbonates homopolymers and block polymers from cyclic monomers, including lactones, epoxides, and carbon dioxide (CO_2_).^11–14^ With the best catalysts, these methods offer excellent control over polymer architectures and enable access to materials derived from renewable or waste feedstocks.^9,15^ However, achieving high molar mass polymers using these methods often requires rigorous monomer purification to eliminate protic impurities, which otherwise limit chain growth by acting as initiators.^16,17^ As a result, the synthesis of low molar mass polymers is typically more straightforward and scalable.^18^ These hydroxyl-terminated oligomers are widely used as precursors in the polyurethane industry, where they are chain-extended with diisocyanates to produce a broad range of materials, including foams, elastomers, adhesives, and coatings.^19–22^

Halatopolymers (from the Greek word *hálas*, meaning salt) are a class of supramolecular materials in which polymers featuring telechelic anionic ligands, typically carboxylates, associate reversibly with divalent metal cations to form physical cross-links.^23^ Originally described by Economy and co-workers in the 1960s, early halatopolymer systems used dicarboxylic acids coordinated with divalent metal salts (e.g., Zn^2+^, Ca^2+^, Mg^2+^), yielding materials with end-groups capable of undergoing rapid exchange and excellent thermal stability.^24^ The dynamic nature of these ionic cross-links allows for thermal reprocessability, offering potential advantages over conventional polyurethanes (PUs), which are typically formed via irreversible reactions between polyols and diisocyanates.^19^ In contrast to isocyanate-based systems, halatopolymer synthesis avoids toxic reagents and may benefit from straightforward synthetic modification of readily accessible polymer precursors.^24^

Recent advances in supramolecular polymer chemistry have leveraged metal–ligand interactions to enhance and tune the thermomechanical performance of high molar mass materials, including block copolymers, and ionomers incorporating terpyridines,^25,26^ catechols,^27^ or pendant carboxylates.^28,29^ These systems have demonstrated dynamic mechanical tunability, improved toughness, and thermal responsiveness.^30^

To date, the application of metal–ligand coordination to low molar mass, linear polymers remains underexplored despite their synthetic accessibility and commercial relevance. Weiss and co-workers have reported a series of metal–carboxylate telechelic poly­(lactic acid) (PLA) plastics in which the metals include Na­(I), Ca­(II), or Y­(III). The glass transition temperature (*T*_g_) increased with the strength of the metal–carboxylate bonds; however, the change in material mechanical properties was not explored.^31^ Subsequently, Kulkarni et al. reported star PLA networked by telechelic carboxylate–sodium interactions. Upon addition of the metal, the networked materials displayed a significant increase in melt elasticity relative to the PLA precuror.^32^ Fraser and co-workers reported the synthesis of well-defined iron­(II)-centered star metalloblock copolymers bearing poly­(ethylene glycol)-poly­(caprolactone)-PLA arms and bipyridine ligands, exhibiting tunable thermomechanical properties and dual degradation pathways via backbone cleavage and metal–ligand dissociation.^33^

Darensbourg and co-workers directly incorporated a well-defined chromium and rhenium complex into poly­(monothiocarbonate) backbones via the copolymerization of carbonyl sulfide and propylene oxide by utilizing the metal carbonyl diols as the initiator.^34^ Recently, Eagan and co-workers reported the synthesis of CO_2_-derived carboxylate-telechelic poly­(propylene carbonate) and poly­(cyclohexene carbonate).^35^ The star polymers were subsequently transformed into β-hydroxy ester vitrimers, exhibiting impressive tensile mechanical properties while retaining reprocessabilty. In another recent report, Weder and co-workers installed 2,6-bis­(1′-methylbenzimidazolyl)­pyridine-zinc complexes between the chain ends of glycol-modified polyethylene terephthalate.^36^ The resulting materials displayed high tensile strength, stiffness, and excellent healability.

Despite the success of recent supramolecular approaches, the thermomechanical properties of the resulting materials are often fixed by the polymer architecture and cannot be readily tuned. Here, we aimed to explore whether telechelic ionomer formation, modulated by the introduction of a monoacid “stopper” or capping ligand, could enable the formation of halatopolymers with tunable effective chain lengths and viscoelastic behavior. By functionalizing low molar mass, bio- and CO_2_-derived ABA block polymers with terminal carboxylic acid groups, we seek to access polymer precursors capable of metal coordination. Coordination with divalent metal salts will be explored to drive supramolecular assembly through reversible ionic cross-linking, offering a strategy for creating reprocessable materials with variable mechanical performances (Figure 1).

**Figure 1:**
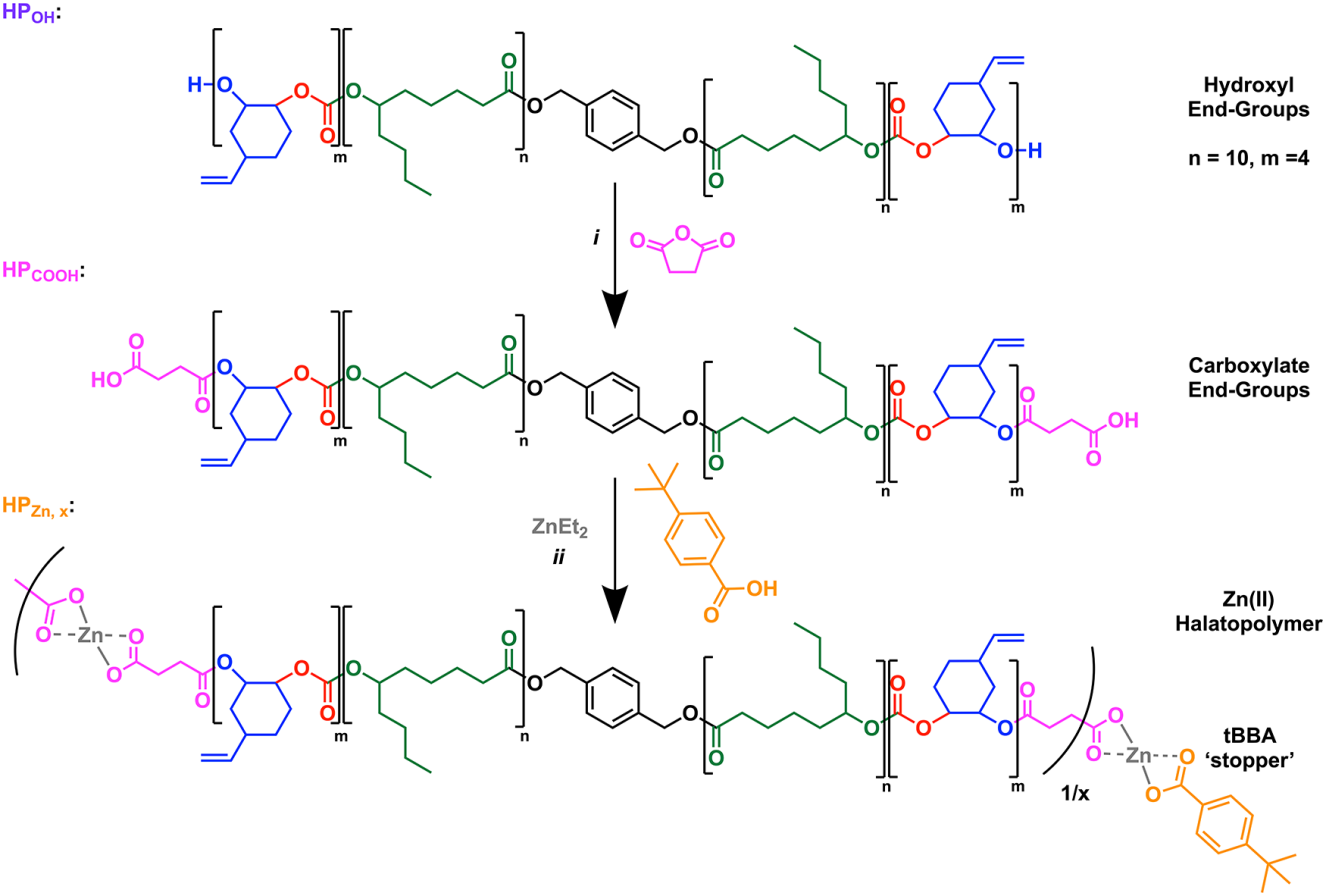
Synthesis of CO_2_ and bioderived halatopolymers by carboxylate end-group installation, followed by Zn­(II) coordination. (i) 4-Dimethylaminopyridine, THF, 40 °C. (ii) THF, r.t. effective chain length (1/*x*) modulated by varying the substoichiometric (*x*) addition of 4-*tert*-butylbenzoic acid (tBBA).

A triblock prepolymer, poly­(vinyl-cyclohexene carbonate-*b*-ε-decalactone-*b*-vinyl-cyclohexene carbonate) (PvCHC-PDL-PvCHC) was prepared in one-pot by switch polymerization catalysis, with a [LCoMg­(OAc)_2_] catalyst (Figure S1).^37–40^ The [Co­(II)­Mg­(II)] catalyst provides high turnover frequencies (TOFs) for both ε-decalactone (εDL) ROP as well as vinyl cyclohexene oxide (vCHO)/CO_2_ ROCOP.^37–40^ Furthermore, the heterodinuclear catalyst is tolerant to high monomer loadings and critically, when used with a diol, shows excellent hydroxyl end-group fidelity.^37–40^ The [Co­(II)­Mg­(II)] catalyst, benzene dimethanol initiator (BDM), ε-DL, and vCHO were dissolved in toluene ([LCoMg­(OAc)_2_]_0_ = 0.35 mM, [LCoMg­(OAc)_2_]_0_:​[BDM]_0_:​[vCHO]_0_:​[ε-DL]_0_ = 1:150:1486:4405). Under a nitrogen atmosphere, the ROP of castor oil derived ε-DL proceeded to high conversion (98%, 80 °C, TOF = 1439 h^–1^).^41,42^ Subsequently, the reaction atmosphere was switched to CO_2_, resulting in a switch from lactone ROP to epoxide/CO_2_ ROCOP and the formation of PvCHC outer blocks with high epoxide conversions (>99%, >99% CO_2_ selectivity, >99% polymer selectivity).

The colorless liquid prepolymer (HP_OH_) was isolated in a high yield (85%) by stirring over Amberchrom 50WX8 (hydrogen form, 100–200 mesh) overnight followed by filtration (twice, through silica) to remove the used catalyst and any residual diol.^37^ The prepolymer was found to possess a molar mass of 6.3 kg mol^–1^ (*Đ*_M_ = 1.26) by SEC (Figure S2). The high loading of diol initiator relative to the initiating acetate coligands resulted in 99% triblock polymer formation and 1% diblock content, which is below the detection limit of SEC or NMR spectroscopy. The prepolymer molar mass, determined by ^1^H NMR spectroscopy (*M*_n,NMR_, Figures S3–S6) was 4.8 kg mol^–1^ as determined by integrating the aromatic resonances of the initiator (BDM) at 7.34 ppm and comparing them with the signals corresponding to the PDL main-chain methylene protons (2.27 ppm) and the terminal vinyl protons of PvCHC (5.76 ppm). This analysis yielded average block degrees of polymerization of 4:10:10:4 for the PvCHC–PDL–PDL–PvCHC prepolymer. The resulting PvCHC content was calculated to be 30 wt %, consistent with the typical block polymer composition range associated with elastomeric behavior.^28^

^1^H DOSY NMR spectroscopy confirmed the formation of a block polymer structure with only a single diffusion coefficient being observed (Figure S7).^43,44^ The expected hydroxy telechelic end-groups were confirmed through the addition of a phospholane reagent to the block polymer and the subsequent observation of a characteristic multiplet between 146 and 147 ppm, in the ^31^P­{^1^H} NMR spectrum, corresponding to the PvCHC–OH end-groups (Figure 2a).^43,44^ The hydroxy telechelic triblock polymer is subsequently referred to as HP_OH_.

**Figure 2:**
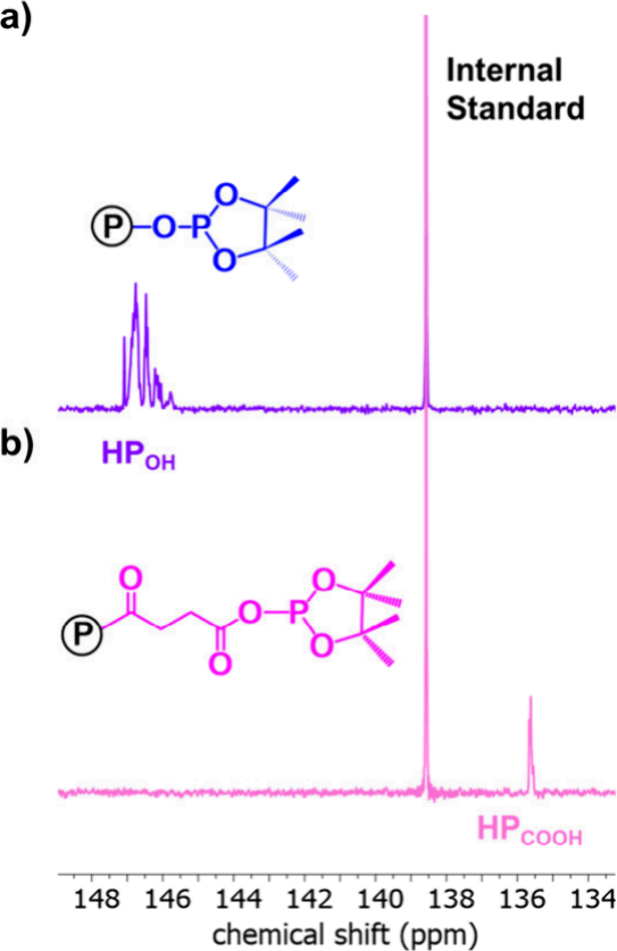
^31^P­{^1^H} NMR spectra showing end-group analysis of (a) hydroxy telechelic (HP_OH_) and (b) carboxylate telechelic (HP_COOH_) triblock polmers. Circled P is the polymer chain.

To install carboxylic acid end-groups, which can later be coordinated to metal ions, HP_OH_ was dissolved in THF (5 wt %) along with succinic anhydride (SA) and 4-dimethylaminopyridine (DMAP) as a catalyst. Initially, a [−OH]_0_:[DMAP]_0_:[SA]_0_ loading of 1:1:40 was employed and the reaction was stirred at 40 °C. The conversion of −OH to −COOH end groups was monitored through the addition of the phospholane reagent and by ^31^P­{^1^H} NMR spectroscopy (Figure S8).^45^ After 7 days, conversion to the acid functionalized polymer plateaued at 94%. Subsequently, the reaction was repeated with a [−OH]_0_:[DMAP]_0_:[SA]_0_ loading of 1:5:40 and full conversion was achieved after 2 days. The carboxylate functionalized triblock polymer (HP_COOH_) was isolated through precipitation in cold methanol (−78 °C, three times), removing excess SA and DMAP, below ^1^H NMR spectroscopy detection limits, in a 59% yield.

End-group analysis confirmed complete end-group functionalization with the loss of the multiplet at 146–147 ppm and the emergence of a signal at 135.6 ppm corresponding to the carboxylic acid end-groups in the ^31^P NMR spectrum (Figure 2b). By comparing the integrals for carboxylic acid end-group signal to an internal standard (bisphenol A) and assuming two acid end-groups per chain, the molar mass of the end-group analysis (*M*_n,EG_) was determined to be 6.2 kg mol^–1^, in good agreement with the value obtained by SEC (*M*_n,SEC_) of HP_OH_. A broad signal at 2.65 ppm in the ^1^H NMR spectrum of HP_COOH_ corresponding to methylene signals of the ring-opened SA was observed (Figure S9). By integrating and comparing the end-group methylene signals to the aromatic signal for the BDM initiator, a BDM:COOH ratio of 1:2 was determined, further indication of complete end-group functionalization.

Halatopolymers were prepared by adding a known amount of diethylzinc (ZnEt_2_) to a THF solution of HP_COOH_ (5 wt %), under a N_2_ atmosphere. The organozinc reagent chosen as the only byproduct upon Zn­(II) coordination is ethane, which can be readily removed to drive the coordination. In all halatopolymer syntheses, a [COOH]:[Zn­(II)] ratio of 1:1 was maintained, while substoichiometric amounts of 4-*tert*-butylbenzoic acid (tBBA) were added in decreasing quantities (Figure 1). We hypothesized that the monofunctional carboxylic acid would act as a chain stopper, limiting the number of triblock polymer chains linked in series via Zn–carboxylate coordination. Although the Zn–carboxylate interactions are expected to be somewhat dynamic and reversible, the extent of supramolecular chain extension could be regulated by the amount of tBBA present, and under such dynamic conditions, transient cyclic species may also form within the equilibrium assembly. Consequently, we anticipated that decreasing the tBBA content should increase the effective chain length, resulting in materials showing an enhanced elastic response and improved dimensional stability. A series of halatopolymers were therefore synthesized with 0.5, 0.2, 0.1, 0.05, and 0.01 equiv of tBBA relative to Zn­(II) (Table 1). The resulting materials are subsequently named HP_Zn,*x*_, where *x* is the equivalents of tBBA incorporated.

**Table 1:** Thermal and Mechanical Properties of CO_2_- and Bio-Derived Halatopolymers

Name	End-Group^a^	eq tBBA^b^	*T*_*g*_, DSC/ °C^c^	*T*_d,5%_ /°C^d^	*η*_0_/Pa s (*T*_ref_ = 25 °C)^e^	*G*′ = *G*″/Hz (*T*_ref_ = 25 °C)^f^	*G*′ = *G*″/°C (ω = 1 Hz)^g^
HP_OH_	OH	0	–39	295	2.2 × 10^3^	-	-
HP_COOH_	COOH	0	–39	247	1.2 × 10^4^	-	-
HP_Zn,0.50_	COOZn	0.5	–41	228	7.6 × 10^5^	5.7 × 10^–2^	45
HP_Zn,0.20_	COOZn	0.2	–41	228	9.3 × 10^5^	2.7 × 10^–2^	47
HP_Zn,0.10_	COOZn	0.1	–44	228	1.5 × 10^6^	1.9 × 10^–2^	49
HP_Zn,0.05_	COOZn	0.05	–40	235	4.0 × 10^6^	1.4 × 10^–2^	51
HP_Zn,0.01_	COOZn	0.01	–42	231	1.1 × 10^7^	2.5 × 10^–3^	56

a End group determined by ^31^P­{^1^H} NMR spectroscopy and 1:1 stoichiometric addition of ZnEt_2_ to acid end-group.

b Relative to Zn­(II).

c Glass transition temperature determined from midpoint of second DSC heating cycle.

d Thermal degradation is the temperature at 5% mass loss, determined by TGA.

e Zero shear viscosity and

f modulus cross over point from oscillatory rheology time–temperature superposition master curve.

g Modulus cross over point from oscillatory rheology temperature ramps.

Once dried, the resulting HP_Zn,*x*_ ionomeric materials were soft but viscoelastic solids, which were significantly more viscous and dimensionally stable than liquid HP_OH_ and HP_COOH_, indicative of successful halatopolymer formation. The synthesis of HP_Zn,0.00_, a halatopolymer containing no tBBA, was attempted multiple times. However, upon drying, the material underwent visible degradation, oxidizing, and discoloring (Figure S10), unlike the materials containing tBBA (even in very low quantities), which remained colorless. This behavior is tentatively attributed to more heterogeneous Zn­(II) coordination environments in the absence of capping ligands. Without tBBA, Zn­(II) ions may engage in a higher proportion of mono- or bidentate bridging with terminal carboxylates, bringing metal centers into closer proximity and potentially forming small coordination clusters. Such local clustering could promote Zn­(II)-catalyzed degradation of ester and carbonate linkages during drying.^46^ The addition of tBBA likely moderates this behavior by both sterically and electronically stabilizing Zn­(II) coordination, disrupting interchain aggregation, and enhancing the thermal stability of the material, underscoring the role of the capping ligand beyond just chain-length control.

The appearance of symmetric and asymmetric metal–carboxylate resonances between 1500 and 1600 cm^–1^ for the halatopolymers by Fourier transform infrared spectroscopy (FTIR) confirmed successful Zn­(II)–carboxylate coordination (Figure 3a). All materials were visibly soluble in THF and chloroform solutions upon coordination with Zn­(II) (5 w/v%). Signals at 8.00 and 7.44 ppm in the ^1^H NMR spectrum of HP_Zn,0.50_ are assigned to the *ortho*- and *meta*-aromatic protons of tBBA, respectively. Furthermore, integration of the aromatic signals confirms the expected tBBA:COOH of 1:2. (Figure S11). SEC analysis of the halatopolymers was not possible, as the samples could not be filtered through 0.2 μm membranes, which is suggestive of aggregation in the solution state.

**Figure 3:**
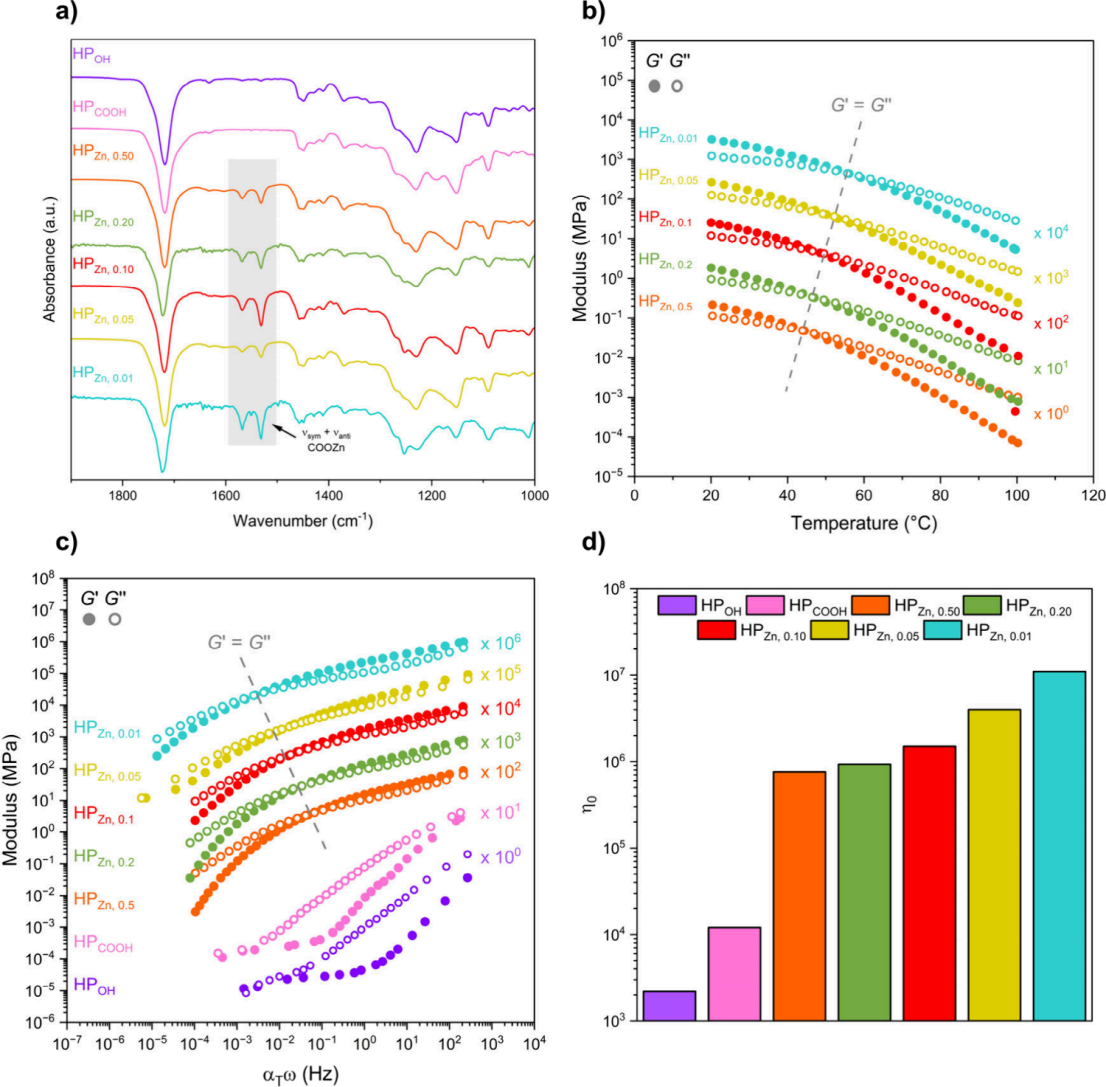
Characterization data for CO_2_- and bioderived halatopolymers (HP_Zn,*x*_). (a) FTIR spectra, shaded region highlighting COOZn stretching bands. (b) Oscillatory rheology temperature ramps and (c) oscillatory rheology time–temperature superposition master curves (moduli offset for clarity). Dashed line marking moduli crossover points. (d) Zero-shear viscosity values.

All of the materials were amorphous with only a single *T*_g_ value observed by differential scanning calorimetry (DSC, Table 1, Figure S12). All *T*_g_ values were ∼ −40 °C and consistent with miscible PvCHC and PDL blocks.^28,37^ Therefore, chain-end coordination, in this molar mass regime, does not increase the block interaction parameter χ sufficiently to drive microphase separation. Thermal gravimetric analysis (TGA) was performed on samples of each material (Table 1 and Figures S13–S19). HP_OH_ displays relatively high thermal stability with 5% mass loss (*T*_d,5%_) occurring at 295 °C. This onset of thermal degredation decreases to 247 °C upon installation of the carboxylic acid end groups in HP_COOH_ and then further to 228–235 °C for each Zn­(II) halatopolymer. The slight decrease in thermal stability for the halatopolymers is attributed to zinc-catalyzed depolymerization of the polyester and polycarbonate samples at high temperatures but does not limit processing.^46,47^

In order to probe the thermomechanical profiles of the halatopolymers and explore the influence of varying halatopolymer chain length and Zn­(II):tBBA ratios, oscillatory rheological temperature ramps were conducted on HP_Zn,0.50_, HP_Zn,0.20_, HP_Zn,0.10_, HP_Zn,0.05_, and HP_Zn,0.01_ over the range of 20–100 °C (Figures 3b and S20–S24). All materials exhibit predominantly rubbery responses at room temperature with the storage modulus greater than the loss modulus (*G*′ > *G*″); however, the temperature at which modulus cross over occurred (*G*′ = *G*″) increased as the stopper end-group (tBBA) content decreased. This finding is indicative of the desired metal:stopper ratio controlling the effective chain length in the halatopolymer. Decreasing the amount of tBBA increases the extent of Zn­(II)–carboxylate-mediated chain coupling, thereby raising the temperature at which the material transitions from the rubbery state to the viscous state.

The construction of time–temperature superposition (TTS) mastercurves (*T*_ref_ = 25 °C) further highlights the tunable nature of Zn­(II)–carboxylate interactions in governing the viscoelastic behavior of the halatopolymers (Figures 3c and S25–S31). As the amount of tBBA is reduced, the crossover point between the *G*′ and *G*″ moduli shifts to lower frequencies, and the material exhibits increasingly solid-like behavior across the accessible range of frequencies. This shift reflects slower relaxation dynamics due to a higher degree of chain coupling through Zn­(II)–carboxylate interactions. By modulating the tBBA content, the average number of chain-ends linked together can be tuned, effectively altering the molar mass of the supramolecular assembly and enabling control over chain dynamics. In contrast, materials lacking Zn­(II) show no rubbery character; the loss modulus remains greater than the storage modulus throughout, indicating an absence of elasticity and consistent with a predominantly viscous response.

The zero-shear viscosity (η_0_), estimated from the low-frequency plateau of the TTS master curves, increases markedly as the proportion of tBBA is reduced (Figures 3d and S32–S38). Installation of carboxylic acid end-groups results in an increase in η_0_ from 2.2 × 10^3^ to 1.2 × 10^4^ Pa s. This almost order of magnitude increase is attributed to the hydrogen bonding between chain ends. With the addition of zinc, η_0_ increased to 7.6 × 10^5^ Pa s for HP_Zn,0.50_ and steadily further up to 1.1 × 10^7^ Pa s for HP_Zn,0.01_. This trend reflects a progressive enhancement in the effective connectivity of polymer chains through Zn­(II)–carboxylate coordination. As fewer Zn­(II) sites are capped by monofunctional tBBA ligands, a greater fraction of Zn­(II) ions link two chain ends, increasing the number of chains incorporated into the halatopolymer supramolecular assemblies and raising the effective molar mass between relaxation events. The result of forming long effective chain lengths is a slower terminal relaxation and a higher zero-shear viscosity. This tunable viscosity behavior directly arises from the molecular-level control over chain coupling, illustrating how modulating the balance between bridging Zn­(II) and capping tBBA interactions enables access to a range of viscoelastic responses.

Rheological creep-recovery experiments (5 kPa for 100 s, 0 kPa for 100 s, 4 cycles, 25 °C) reveal that the dimensional stability of the halatopolymers improves significantly with zinc coordination (Figure 4). HP_OH_ exceeds 1.0 × 10^6^ % strain by the final loading cycle, while the hydrogen bonded end-groups of HP_COOH_ limit deformation to 1.8 × 10^5^ %. Both viscous materials exhibited no recovery during the 0 kPa cycles indicative of no elastic behavior. In contrast, the Zn­(II) halatopolymers exhibit far less creep under the applied force (<160%). Samples with lower tBBA exhibit less creep under constant stress, and all show improved recovery upon unloading, with HP_Zn,0.01_ exhibiting a maximum strain of 45% on the fourth cycle, indicative of increased resistance to permanent deformation. This behavior is attributed to the higher average number of chains coupled through Zn­(II)–carboxylate interactions at reduced tBBA levels, as the material’s ability to resist flow under stress is reinforced. These results underscore how controlling the degree of chain coupling via tBBA content enables tuning of dimensional stability and elastic recovery. This strategy demonstrates a modular approach to tuning viscoelasticity in supramolecular polymer networks, offering a straightforward method to control the properties of the resulting oxygenated elastomers and adaptive soft materials.

**Figure 4:**
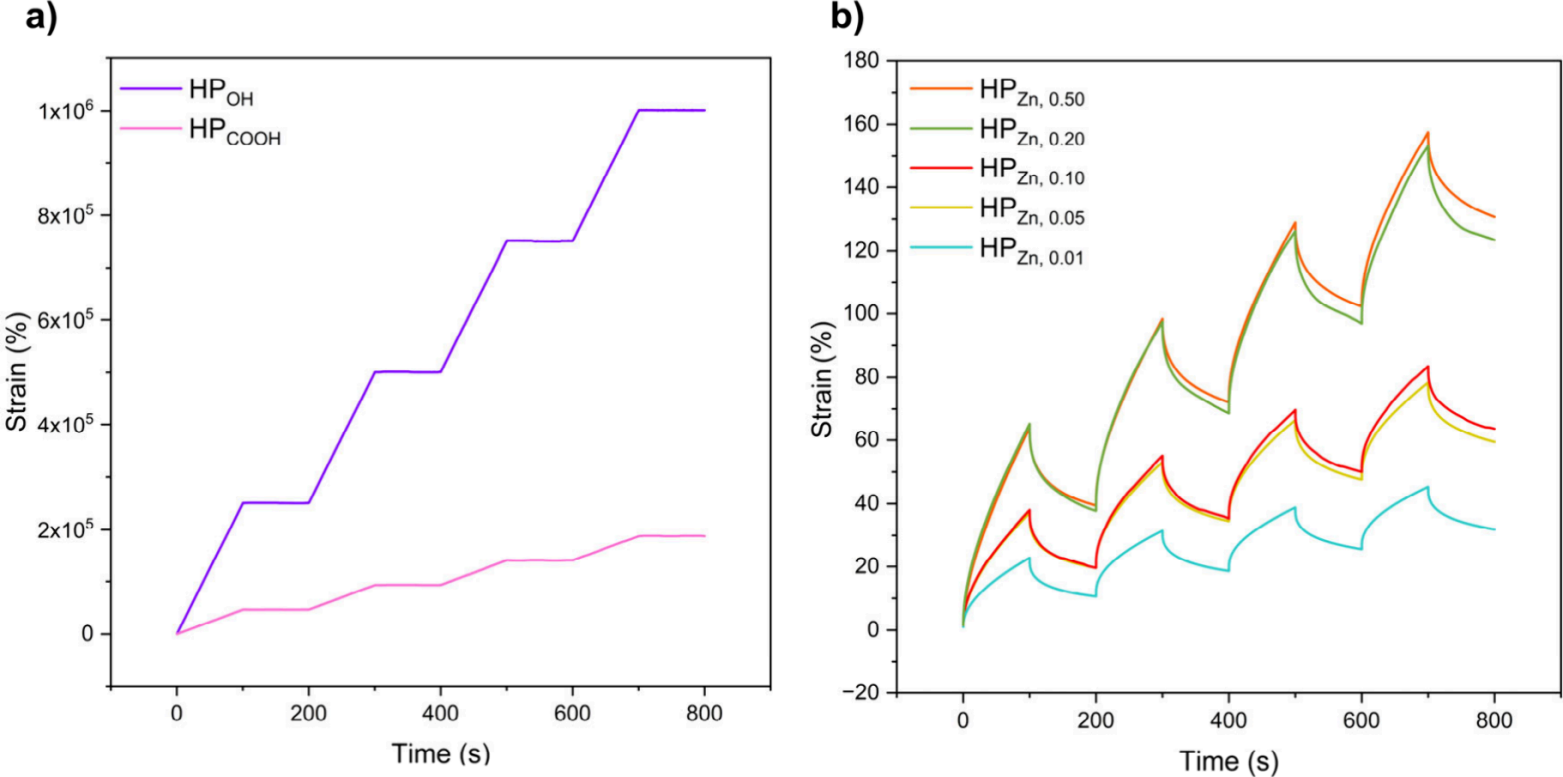
Creep-recovery experiments at 25 °C, 5 kPa for 100 s, 0 kPa for 100s, and 4 cycles for (a) HP_OH_ and HP_COOH_ and (b) Zn­(II) halatopolymers.

## Supplementary Material



## Data Availability

The data for this article are available at: https://dx.doi.org/10.5287/ora-vjbnp9eqr.

## Notes

Experimental and instrument details, polymer synthesis, characterization, and additional mechanical property measurements (PDF)
