# Supplementary material for: Block CO2–Polycarbonates: Tunable Chain Extension with Zn(II) Carboxylates
Source: ACS Macro Lett. 2025 Nov 5;14(11):1755–61. doi: 10.1021/acsmacrolett.5c00639 (PMC12632168; doi:10.1021/acsmacrolett.5c00639)
Supplement: Supplementary file 1 [file mz5c00639_si_001.pdf]

Supporting Information:

Block CO_2-Polycarbonates: Tuneable Chain Extension with Zn(II) Carboxylates

Kam C. Poon, Chang Gao, Diego A. Resendiz-Lara, Mantas Drelingas and Charlotte K. Williams*

Chemistry Research Laboratory, Department of Chemistry, University of Oxford, Oxford, OX1 3TA, U.K.

E-mail: charlotte.williams@chem.ox.ac.uk

Contents

Experimental Details 4

Reagents and Methods 5

Figure S1: Synthesis of poly(carbonate-ester-carbonate), HP_{OH}. (i) ε-DL ROP, 80˚C, [LCoMg(OAc)$_2$ with BDM as the initiator, where $[Cat.]_0:[BDM]_0:[vCHO]_0:[ε\text{-}DL]_0$ = 1:150:1486:4405, $[LCoMg(OAc)_2]_0$ = 0.35 mM, toluene. (ii) CO_2/vCHO ROCOP, CO_2 (1 bar), 80 ˚C. PvCHC-PDL-PvCHC (HP_{OH}): $M_{n,\,SEC}$ = 6.3 kg mol^{-1} ($Đ_M$ = 1.26), 30 wt% PvCHC by ^{1}H NMR spectroscopy.[3] 7

Figure S2: SEC (THF, 1 mL min^{-1}) traces for HP_{OH} and HP_{COOH}. 8

Figure S3: ^{1}H NMR (400 MHz, $CDCl_3$) spectrum of HP_{OH}.[3] 9

Figure S4: ^{1}H COSY NMR (400 MHz, $CDCl_3$) spectrum of HP_{OH}.[3] 9

Figure S5: ^{13}C{^{1}H} NMR (150 MHz, $CDCl_3$) spectrum of HP_{OH}.[3] 10

Figure S6: ^{1}H-^{13}C HSQC NMR (400 MHz, $CDCl_3$) spectrum of HP_{OH}.[3] 10

Figure S7: ^{1}H DOSY NMR (400 MHz, $CDCl_3$) spectrum of HP_{OH}.[3] 11

Figure S8: SA end-capping conversion monitored by ^{31}P{^{1}H} NMR end-group test of aliquots. 11

Figure S9: ^{1}H NMR (400 MHz, $CDCl_3$) spectrum of HP_{COOH}. 12

Figure S10: Digital photograph of a) clear and colourless $HP_{Zn,\,0.05}$ and b) discoloured $HP_{Zn,\,0.00}$. c) ^{31}P NMR end-group test of $HP_{Zn,\,0.00}$ showing a new signal at 147 ppm, characteristic of hydroxyl end-groups (6-hydroxydecanoic acid or poly(ε-decalactone) chain ends), consistent with Zn(II)-catalyzed degradation. 12

Figure S11: ^{1}H NMR (400 MHz, $CDCl_3$) spectrum of $HP_{Zn,\,0.50}$. 13

Figure S12: Differential Scanning Calorimetry (DSC) second heating cycle traces of HP_{OH}, HP_{COOH}, $HP_{Zn,\,0.50}$, $HP_{Zn,\,0.20}$, $HP_{Zn,\,0.10}$, $HP_{Zn,\,0.05}$, $HP_{Zn,\,0.01}$. 13

Figure S13: TGA profile for HP_{OH}. 14

Figure S14: TGA profile for HP_{COOH}. 14

Figure S15: TGA profile for $HP_{Zn,\,0.50}$. 15

Figure S16: TGA profile for $HP_{Zn,\,0.20}$. 15

Figure S17: TGA profile for $HP_{Zn,\,0.10}$. 16

Figure S18: TGA profile for $HP_{Zn,\,0.05}$. 16

Figure S19: TGA profile for for $HP_{Zn,\,0.01}$. 17

Figure S20: Rheological temperature ramp for $HP_{Zn,\,0.50}$. 17

Figure S21: Rheological temperature ramp for $HP_{Zn,\,0.20}$. 18

Figure S22: Rheological temperature ramp for $HP_{Zn,\,0.10}$. 18

Figure S23: Rheological temperature ramp for $HP_{Zn,\,0.05}$. 19

Figure S24: Rheological temperature ramp for $HP_{Zn,\,0.01}$. 19

Figure S25: TTS mater curve for HP_{OH}. 20

Figure S26: TTS mater curve for HP_{COOH}. 20

Figure S27: TTS mater curve for $HP_{Zn, 0.50}$. 21
Figure S28: TTS mater curve for $HP_{Zn, 0.20}$. 21
Figure S29: TTS mater curve for $HP_{Zn, 0.10}$. 22
Figure S30: TTS mater curve for $HP_{Zn, 0.05}$. 22
Figure S31: TTS mater curve for $HP_{Zn, 0.01}$. 23
Figure S32: Plot of shear viscosity vs frequency (Hz) obtained from TTS master curves and projection of the plateau viscosity to zero-shear for HP_{OH}. Note: The complex viscosity does not fully plateau in the time–temperature superposition, so the reported zero-shear viscosity is likely a slight underestimation. 23
Figure S33: Plot of shear viscosity vs frequency (Hz) obtained from TTS master curves and projection of the plateau viscosity to zero-shear for HP_{COOH}. Note: The complex viscosity does not fully plateau in the time–temperature superposition, so the reported zero-shear viscosity is likely a slight underestimation. 24
Figure S34: Plot of shear viscosity vs frequency (Hz) obtained from TTS master curves and projection of the plateau viscosity to zero-shear for $HP_{Zn, 0.50}$. 24
Figure S35: Plot of shear viscosity vs frequency (Hz) obtained from TTS master curves and projection of the plateau viscosity to zero-shear for $HP_{Zn, 0.20}$. 25
Figure S36: Plot of shear viscosity vs frequency (Hz) obtained from TTS master curves and projection of the plateau viscosity to zero-shear for $HP_{Zn, 0.10}$. 25
Figure S37: Plot of shear viscosity vs frequency (Hz) obtained from TTS master curves and projection of the plateau viscosity to zero-shear for $HP_{Zn, 0.05}$. 26
Figure S38: Plot of shear viscosity vs frequency (Hz) obtained from TTS master curves and projection of the plateau viscosity to zero-shear for $HP_{Zn, 0.01}$. 26
References 27

Experimental Details

NMR Spectroscopy. ^{1}H and $^{31}P\{^{1}H\}$ NMR spectra were obtained using a Bruker AVIII HD 400 NMR spectrometer. ^{13}C $\{^{1}H\}$ NMR spectra were obtained using a Bruker Avance III AVD500 NMR spectrometer. ^{1}H DOSY spectra were obtained using a Bruker NEO600 NMR spectrometer.

Size Exclusion Chromatography (SEC). Polymers (2-5 mg) dissolved in THF. Samples were passed through 0.2 µm PTFE filters prior to analysis. Analysis was carried out on a Shimadzu LC-20AD instrument, equipped with a Refractive Index (RI) detector and two PSS SDV 5 µm linear M columns. HPLC grade THF was used as the eluent at 1.0 mL/min at 30 °C. Monodisperse polystyrene standards were used for calibration.

Phosphorus End-Group Tests. Polymer samples (20 mg) were dissolved in $CDCl_3$ (0.5 mL) and a solution (40 µL) containing $Cr(acac)_3$ (5.5 mg) and internal standard, bisphenol A (400 mg) in pyridine (10 mL), followed by 40 µL of 2-chloro-4,4,5,5-tetramethyl dioxaphospholane.[1]

Differential Scanning Calorimetry (DSC). Recorded for purified polymer samples of homopolymers and terpolymers were measured using a DSC25 (TA Instruments). A sealed, empty crucible was used as a reference, and the DSC was calibrated using sapphire and indium. Samples were heated from -80 °C to 150 °C, at a rate of 10 °C min^{-1} under N_2 flow (80 mL min^{-1}) followed by a 5-minute isotherm at 150 °C to erase thermal history. Samples were subsequently cooled to -80 °C, at a rate of 10 °C min^{-1}, and kept at -80 °C for a further 5 minutes, followed by a heating-cooling procedure from -80 °C to 150 °C, at a rate of 10 °C min^{-1}. Each sample was analysed over two heating-cooling cycles. Glass transition temperatures (T_g) are reported as the midpoint of the transition taken from the second heating cycle.

Thermogravimetric Analysis (TGA). Measured using a TGA5500 system (TA Instruments). Samples were heated from 30 °C to 600 °C, at a rate of 5 °C min^{-1}, under N_2 flow (100 cm^3 min^{-1}).

Oscillatory Rheology. Conducted on an ARES-G2 (TA Instruments) using polymer samples between 8 mm stainless steel plates. For oscillatory temperature ramps, samples were heated from 30 °C to 100 °C, at a rate of 3 °C min^{-1}, with a frequency of 1 Hz, and 1% strain amplitude. For time-temperature superposition master curves, samples were heated from 20 to 100 °C, with frequency sweeps from 0.1 to 100 Hz, collected at 10 °C intervals (1% strain amplitude). For creep-recovery experiments, stress control conditioning experiments were conducted at 30 °C in the linear viscoelastic region as determined by amplitude sweeps. 5 kPa of stress was applied to the samples for 100 s followed by a period of 0 kPa for 100 s, and the stain (creep) exhibited monitored, this was repeated a further 3 times.

Reagents and Methods

The macrocyclic ligand, H2L, was synthesized following a procedure reported previously.[2] Magnesium bis(1,1,1,3,3,3-hexamethyldisilazan-2-ide) (97%) and cobalt acetate (98%) were purchased from Sigma-Aldrich. Cobalt acetate was used as received and magnesium bis(1,1,1,3,3,3-hexamethyldisilazan-2-ide) was recrystallized from hexanes. Solvents used for synthesis and polymerization were collected from a solvent purification system (SPS), degassed with three freeze-pump-thaw cycles, and stored over 4 Å molecular sieves, under an inert atmosphere. 4-Vinyl-1-cyclohenxene 1,2-epoxide (98 %) (vCHO) was purchased from Sigma-Aldrich, dried by stirring over CaH2, followed by fractional distillation at 60 °C and 18 mbar. It was stored under a nitrogen atmosphere. ε-Decalactone (ε-DL) was dried over CaH2, followed by fractional distillation at 115 °C under reduced pressure, and kept under a nitrogen atmosphere. 1,4-Benzenedimethanol (BDM) was recrystallized from toluene and stored under inert atmosphere. Research-grade carbon dioxide was dried through a Drierite column and two additional drying columns (Micro Torr, Model number: MC1-804FV) in series before use. Diethyl zinc (100%) and 4-*tert*-Butylbenzoic acid (100%) were both purchased from Sigma-Aldrich and used as received.

Synthesis of [LCoMg(OAc)$_2$] Catalyst[2]

Mg(N(SiMe$_3$)$_2$)$_2$ (0.44 g, 0.13 mmol) and Co(OAc)$_2$ (0.23 g, 0.13 mmol) was added to a solution of H$_2$L (0.70 g, 0.13 mmol) in THF (6 mL) and heated to 100 °C in a sealed Schlenk tube for 16 h. The solvent was removed, in vacuo, and the solid washed with pentane (3 x 10 mL) to afford the product as a pale pink solid. Yield: 0.41 g (43 %). MS (MALDI-ToF): m/z 692.31 [LMgCo(OAC)]$^+$.

Procedure for HP$_{OH}$ Synthesis[3]

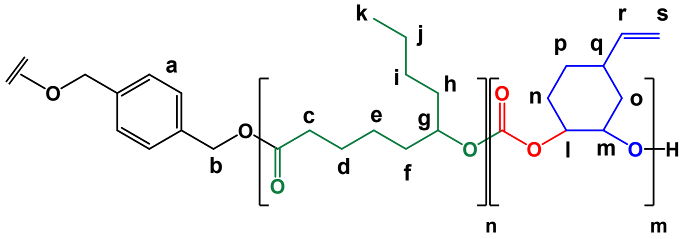

[LCoMg(OAc)$_2$] (250 mg, 0.33 mmol), benzene dimethanol (6.89 g, 50 mmol), 4-Vinyl-1-cyclohenxene 1,2-epoxide (46 g, 371 mmol) and ε-decalactone (187 g, 1098 mmol) were dissolved in toluene (157 mL) and stirred at 80 °C for 3 h (98 % PDL conversion). Next, the reaction was placed under 1 bar of CO_2 in a glass round bottomed Schlenk flask, heated to 80 °C and stirred for 96 h (>99% PvCHC conversion). The reaction mixture was quenched by exposure to air. The triblock copolymer was isolated and purified by dilution in THF, stirring with Amberchrom™ 50WX8 (hydrogen form, 100-200 mesh) overnight, filtration (twice, through silica) to remove residual catalyst and any diol. Following concentration *in vacuo*, the colorless polymer was collected with 85% yield, 204 g (HP$_{OH}$). ^{1}H NMR (400 MHz, CDCl$_3$) δ 7.34 (s, 4H, a), 5.76 (m, 1H, r), 5.04 (m. 5H, b+s), 4.85 (m, 1H, g), 4.77 (br s, 2H, l+m), 2.43 (br s, 1H, q), 2.27 (t, 2H, c), 1.85-1.29 (br m, 20H, d+e+f+g+h+i+j+n+o+p), 0.88 (t, 3H, k). Integrals given are for a single repeat unit of each block. $M_{n,SEC}$ = 6.3 [1.26]

Procedure for HP_{COOH} Synthesis

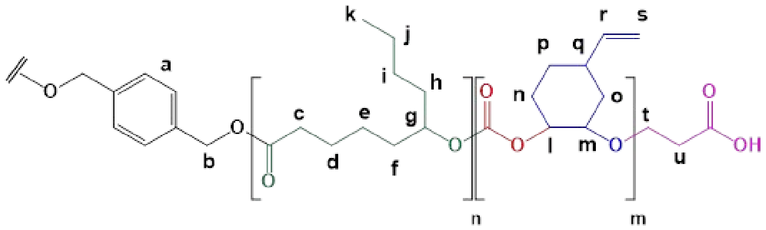

HP_{OH} was dissolved in dry THF (1 g, 0.2 mmol,10 mL). Succinic anhydride (SA, 800 mg, 8 mmol) and 4-dimethylaminopyridine (DMAP, 115 mg, 1.0 mmol) were added to the polymer solution. The reaction mixture was stirred at 40 °C for 72 h. The end-capped polymer was isolated by precipitation in cold methanol (-78 ˚C, three times) and dried *in vacuo* with a 59% yield, 0.60 g. ^{1}H NMR (400 MHz, $CDCl_3$) δ 7.34 (s, 4H, a), 5.76 (m, 1H, r), 5.04 (m. 5H, b+s), 4.85 (m, 1H, g), 4.77 (br s, 2H, l+m), 2.64 (br s, 4H, t+u), 2.43 (br s, 1H, q), 2.27 (t, 2H, c), 1.85-1.29 (br m, 20H, d+e+f+g+h+i+j+n+o+p), 0.88 (t, 3H, k). Integrals given are for a single repeat unit of each block. $M_{n,\,SEC}$ = 7.4 [1.22]

Procedure for $HP_{Zn,\,x}$ Synthesis

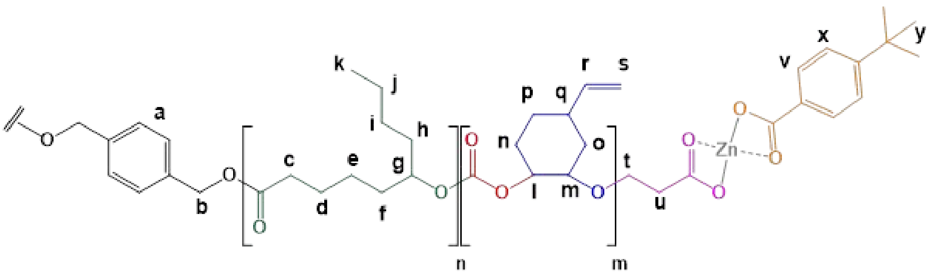

All halatopolymer were synthesised under anaerobic conditions. HP_{COOH} (50 mg, 0.008 mmol) was dissolved in dry THF (1 mL). 4-*tert*-Butyl-benzoic acid (tBBA) was added from a THF stock solution (1 mg in 5 mL, 1.65 mL, 0.004 mmol) and a $ZnEt_2$ THF stock solution (10 mg in 5 mL, 0.27 mL, 0.008 mmol) was slowly added dropwise to the polymer solution. The reaction mixture was stirred at room temperature for 1 h, solvent cast, and dried *in vacuo* at 60 ˚C with a 98 % yield, 50 mg. ^{1}H NMR (400 MHz, $CDCl_3$) δ 8.02 (br s, 2H, v), 7.43 (br s, 2H, x) 7.34 (s, 4H, a), 5.76 (m, 1H, r), 5.04 (m. 5H, b+s), 4.85 (m, 1H, g), 4.77 (br s, 2H, l+m), 2.64 (br s, 4H, t+u), 2.43 (br s, 1H, q), 2.27 (t, 2H, c), 1.85-1.29 (br m, 20H, d+e+f+g+h+i+j+n+o+p+y), 0.88 (t, 3H, k). Integrals given are for a single repeat unit of each block.

Table S1: Summary of halatopolymer reagent quantities

Material	HP_{COOH}	$ZnEt_2$ Stock Solution	tBBA Stock Solution
$HP_{Zn,\,0.50}$	49.44 mg	0.27mL	1.65 mL
$HP_{Zn,\,0.20}$	48.70 mg	0.26 mL	0.65 mL
$HP_{Zn,\,0.10}$	49.41 mg	0.27 mL	0.33mL
$HP_{Zn,\,0.05}$	53.60 mg	0.26 mL	0.18 mL
$HP_{Zn,\,0.01}$	49.55 mg	0.27 mL	0.03 mL

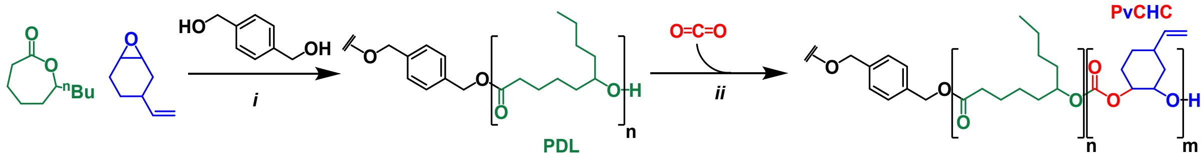

Figure S1: Synthesis of poly(carbonate-ester-carbonate), HP_{OH}. (i) ε-DL ROP, 80˚C, $[LCoMg(OAc)_2$ with BDM as the initiator, where $[Cat.]_0:[BDM]_0:[vCHO]_0:[ε\text{-}DL]_0$ = 1:150:1486:4405, $[LCoMg(OAc)_2]_0$ = 0.35 mM, toluene. (ii) CO_2/vCHO ROCOP, CO_2 (1 bar), 80 ˚C. PvCHC-PDL-PvCHC (HP_{OH}): $M_{n,\ SEC}$ = 6.3 kg mol^{-1} ($Đ_M$ = 1.26), 30 wt% PvCHC by ^1H NMR spectroscopy.[3]

The residual diblock copolymer content (1.316%) represents a theoretical maximum estimated from the molar ratio of difunctional benzene dimethanol (BDM) and monofunctional acetate ligands on the catalyst. Both species can serve as initiators or chain transfer agents during polymerization. As each catalyst molecule bears two acetate co-ligands and the polymerization employs 150 equivalents of BDM, the maximum proportion of chains initiated from acetate is calculated as 2 / (150 + 2) = 1.316%. In practice, the actual diblock content is likely much lower because residual diols present in the vCHO monomer, formed *via* hydrolysis of the epoxide by trace water or CO_2, also act as bifunctional initiators. Experimentally, ^1H NMR spectroscopy of the resulting polymer shows only resonances attributable to BDM-derived initiating groups; no acetate end-group signals are detected. These results indicate that any diblock copolymer species in HP_{OH} are present at levels below the detection limit of NMR spectroscopy, although a small fraction is theoretically expected.

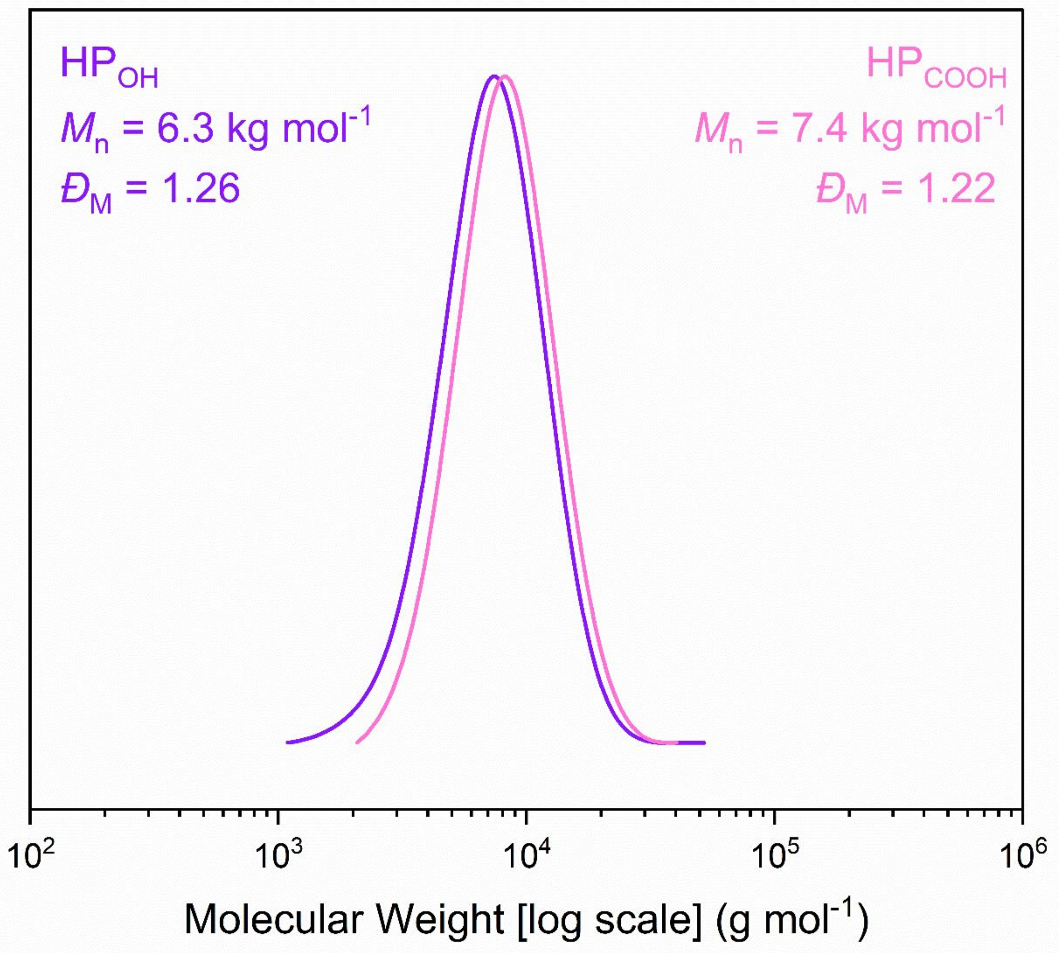

Figure S2: SEC (THF, 1 mL min^{-1}) traces for HP$_{OH}$ and HP$_{COOH}$.

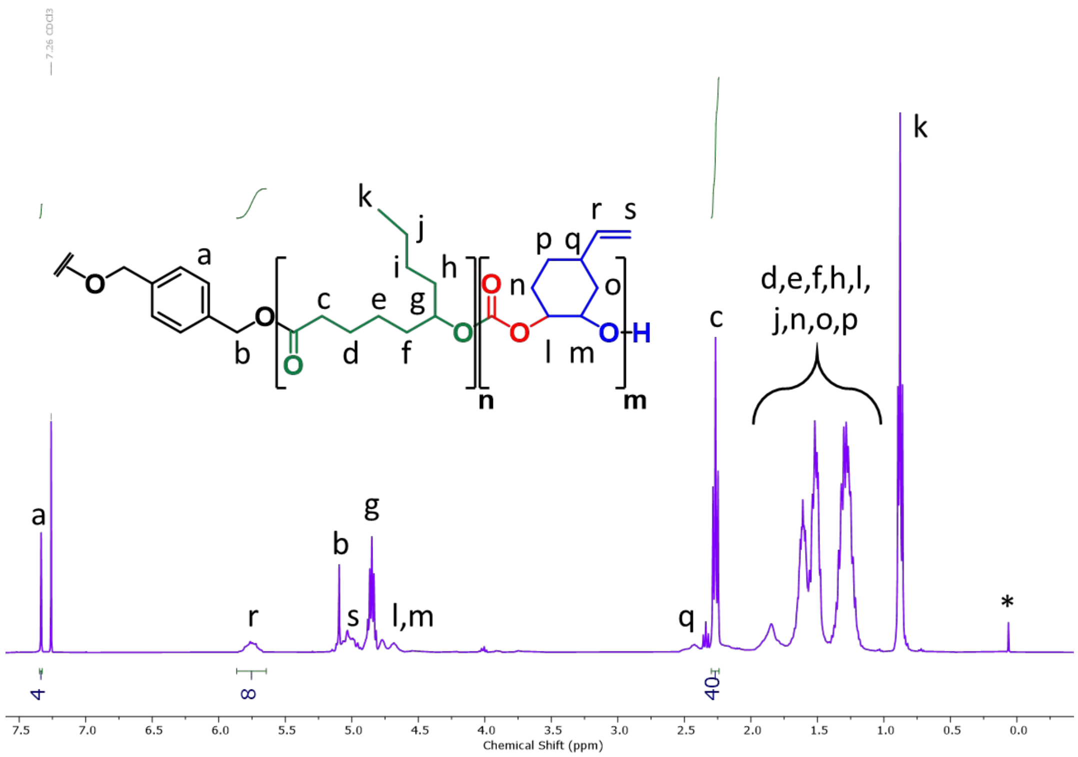

Figure S3: ^{1}H NMR (400 MHz, $CDCl_3$) spectrum of HP_{OH}.[3]

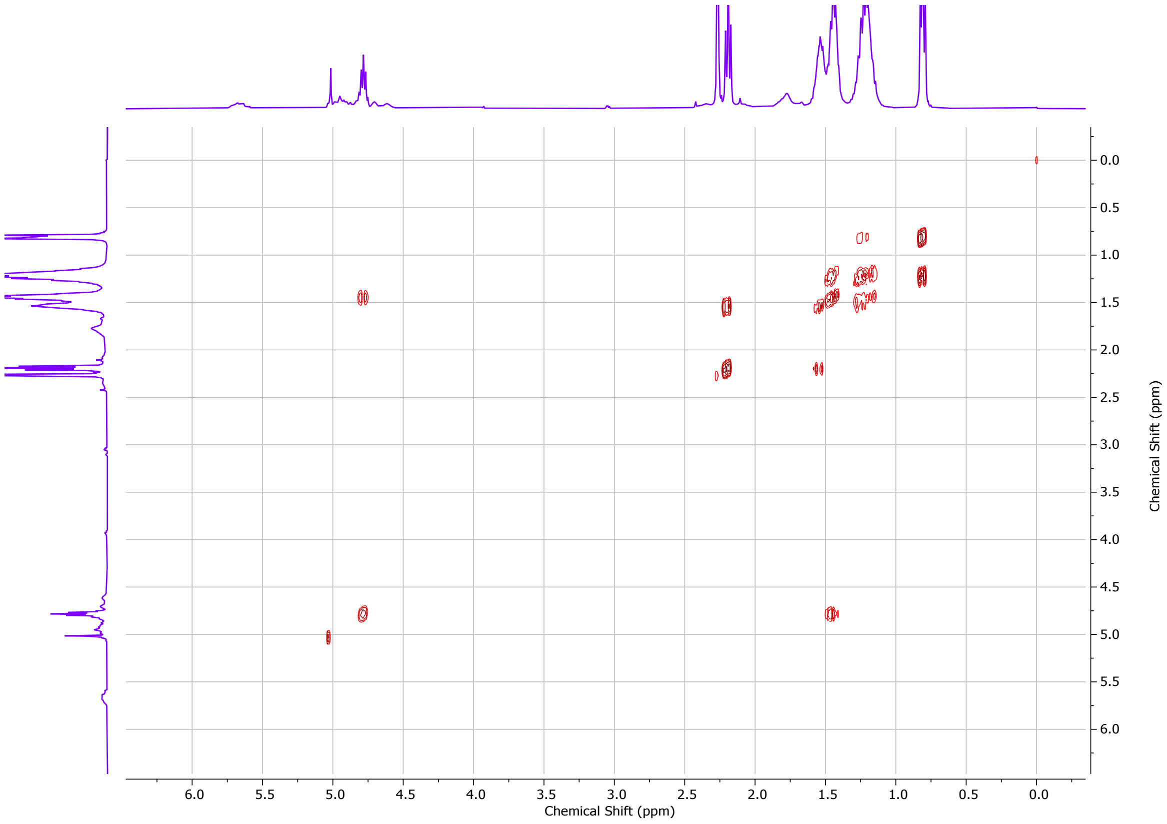

Figure S4: ^{1}H COSY NMR (400 MHz, $CDCl_3$) spectrum of HP_{OH}.[3]

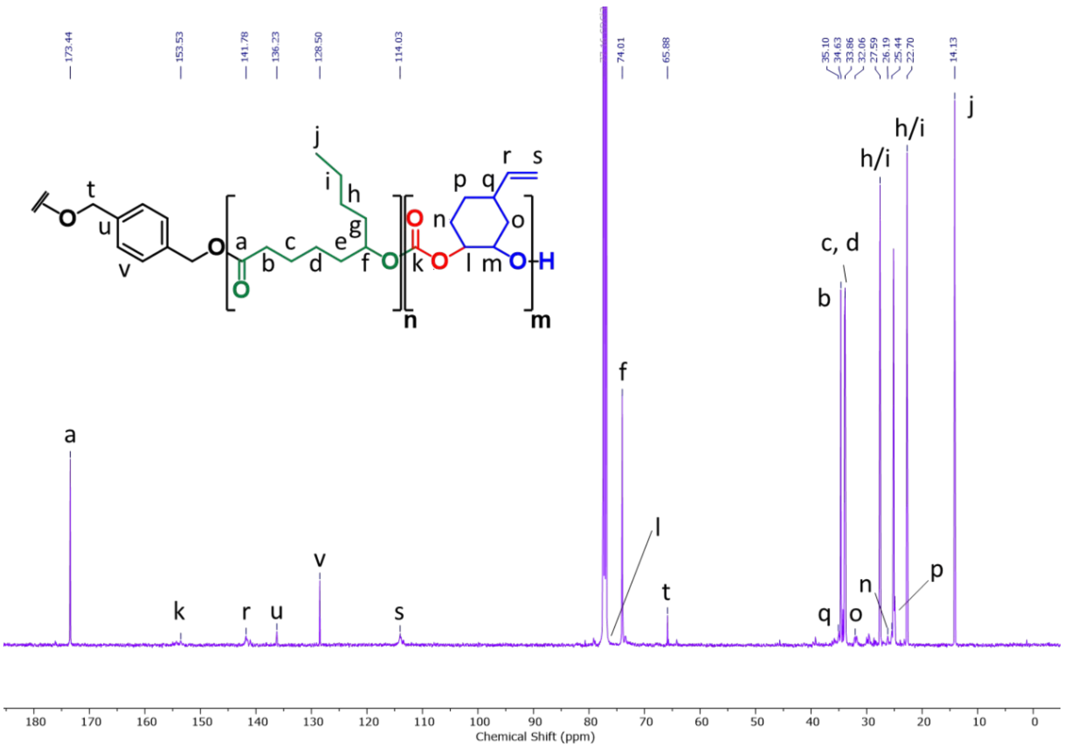

Figure S5: $^{13}C\{^{1}H\}$ NMR (150 MHz, $CDCl_3$) spectrum of HP_{OH}.[3]

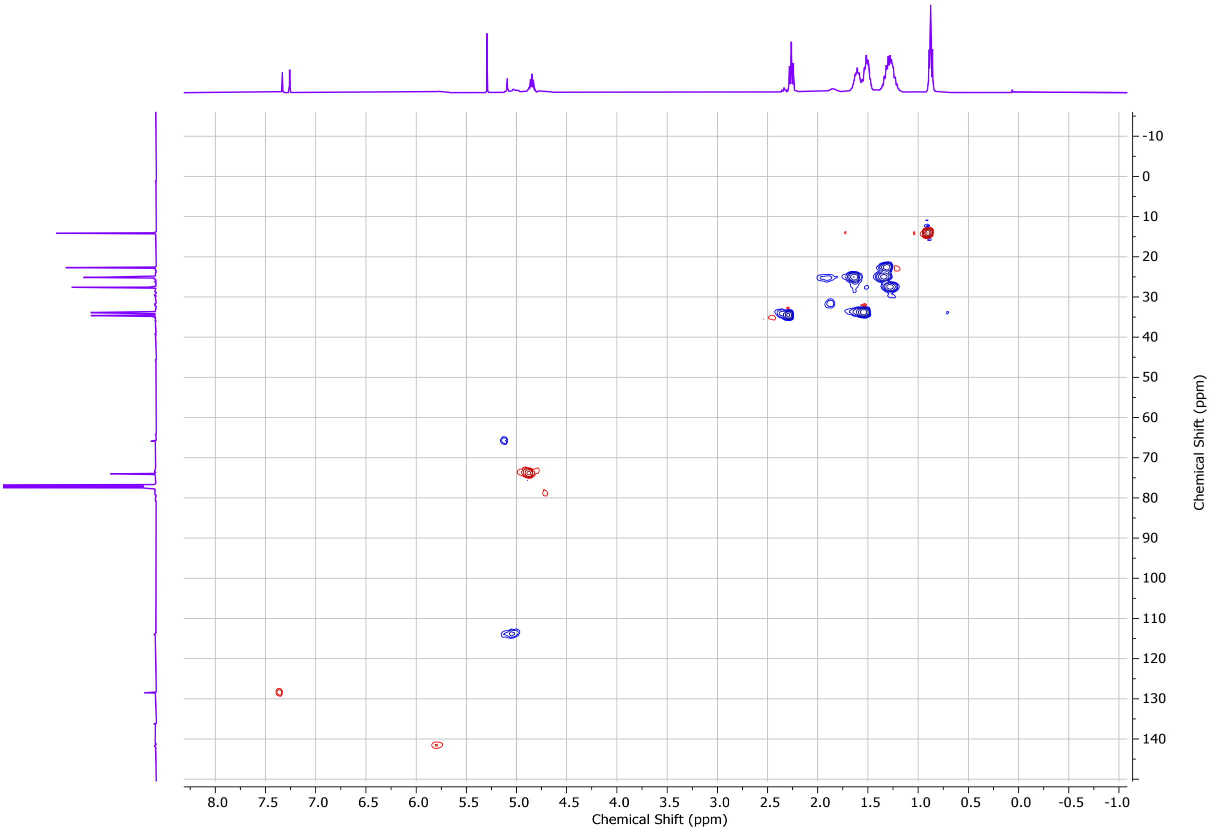

Figure S6: ^{1}H-^{13}C HSQC NMR (400 MHz, $CDCl_3$) spectrum of HP_{OH}.[3]

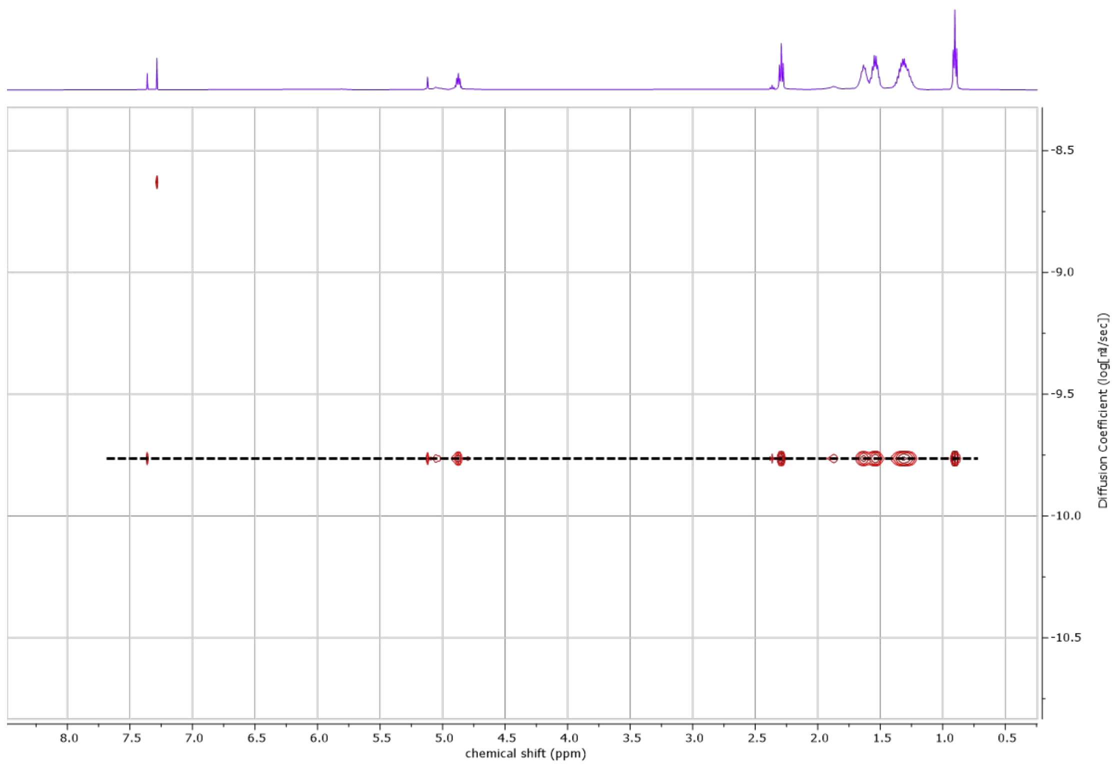

Figure S7: ^{1}H DOSY NMR (400 MHz, $CDCl_3$) spectrum of HP_{OH}.[3]

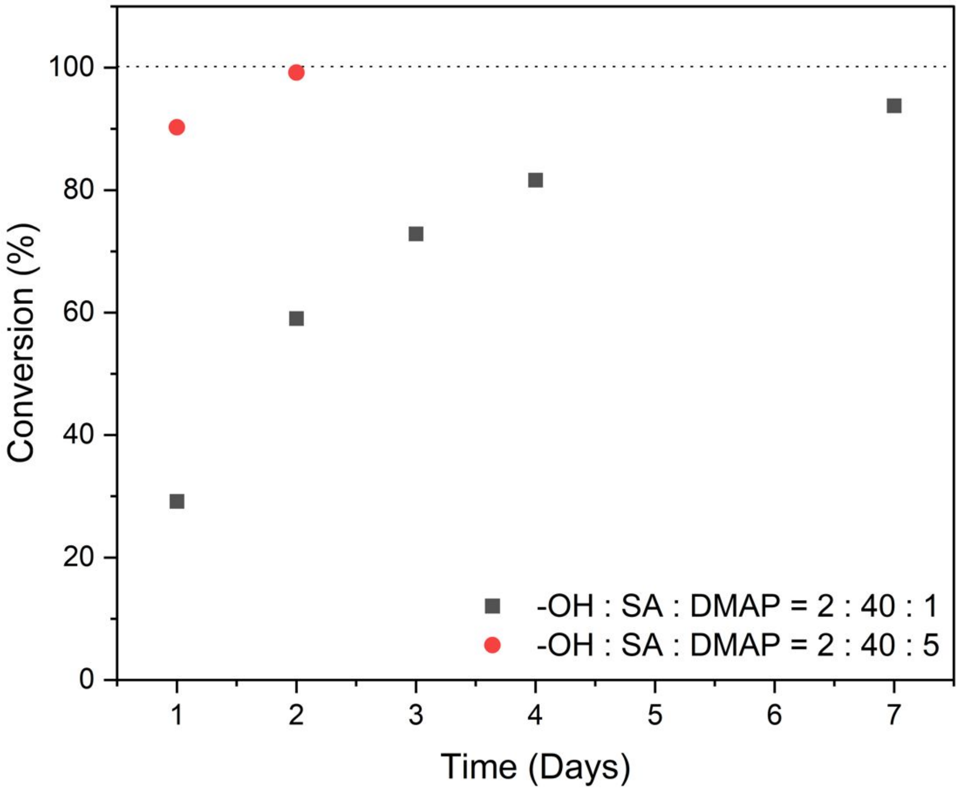

Figure S8: SA end-capping conversion monitored by $^{31}P\{^{1}H\}$ NMR end-group test of aliquots.

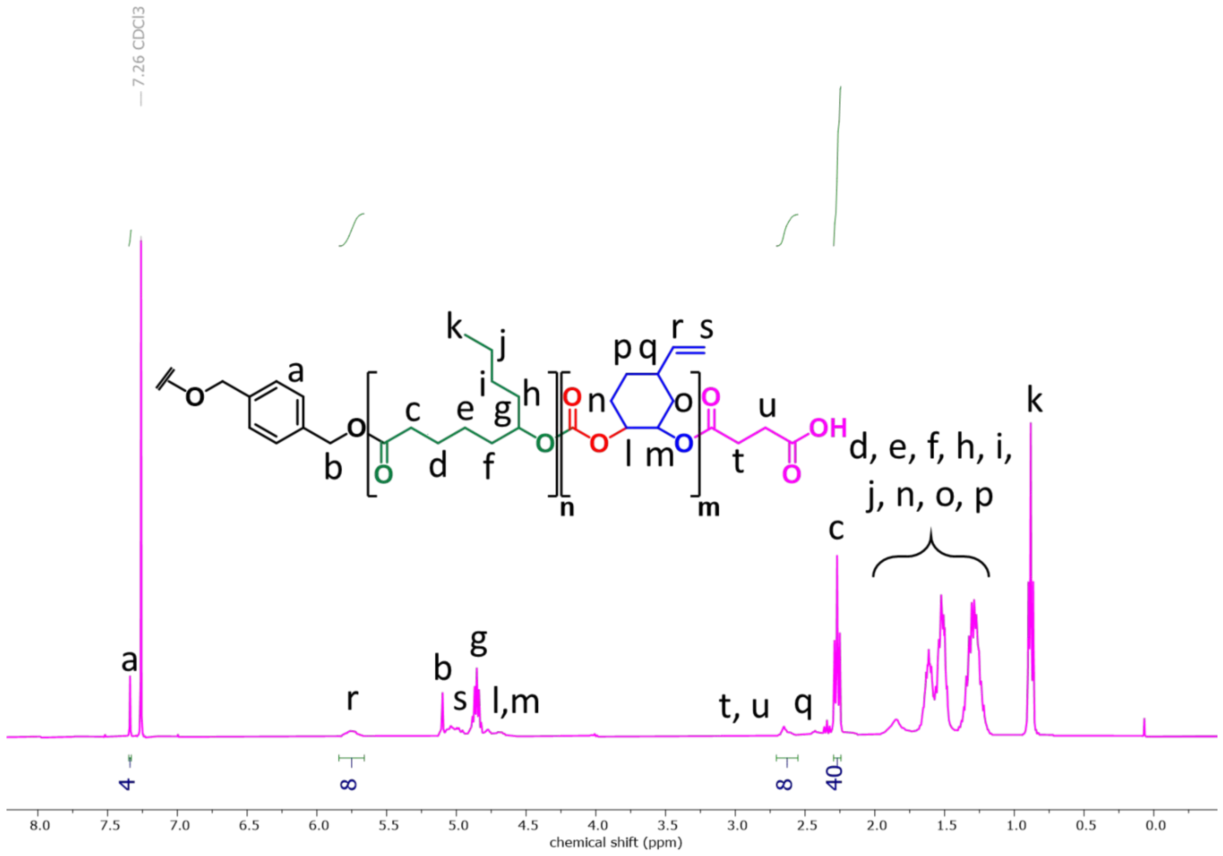

Figure S9: ^{1}H NMR (400 MHz, $CDCl_3$) spectrum of HP_{COOH}.

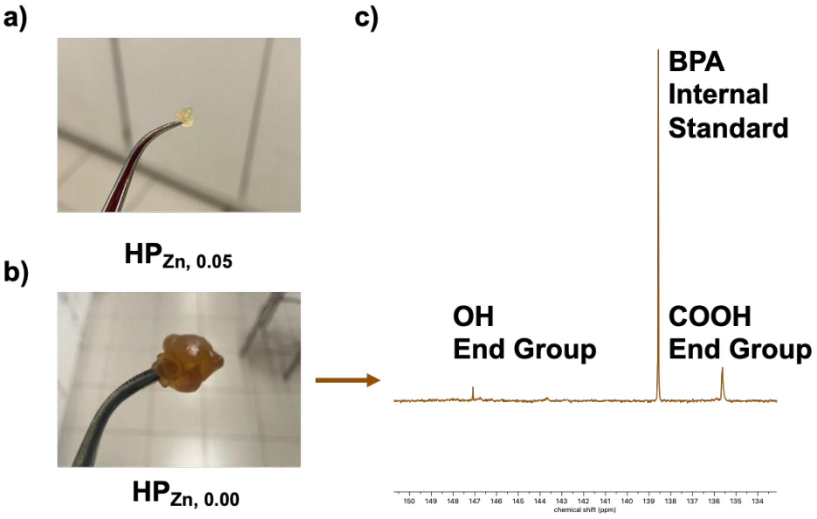

Figure S10: Digital photograph of a) clear and colourless $HP_{Zn,\ 0.05}$ and b) discoloured $HP_{Zn,\ 0.00}$. c) ^{31}P NMR end-group test of $HP_{Zn,\ 0.00}$ showing a new signal at 147 ppm, characteristic of hydroxyl end-groups (6-hydroxydecanoic acid or poly(ε-decalactone) chain ends), consistent with Zn(II)-catalyzed degradation.

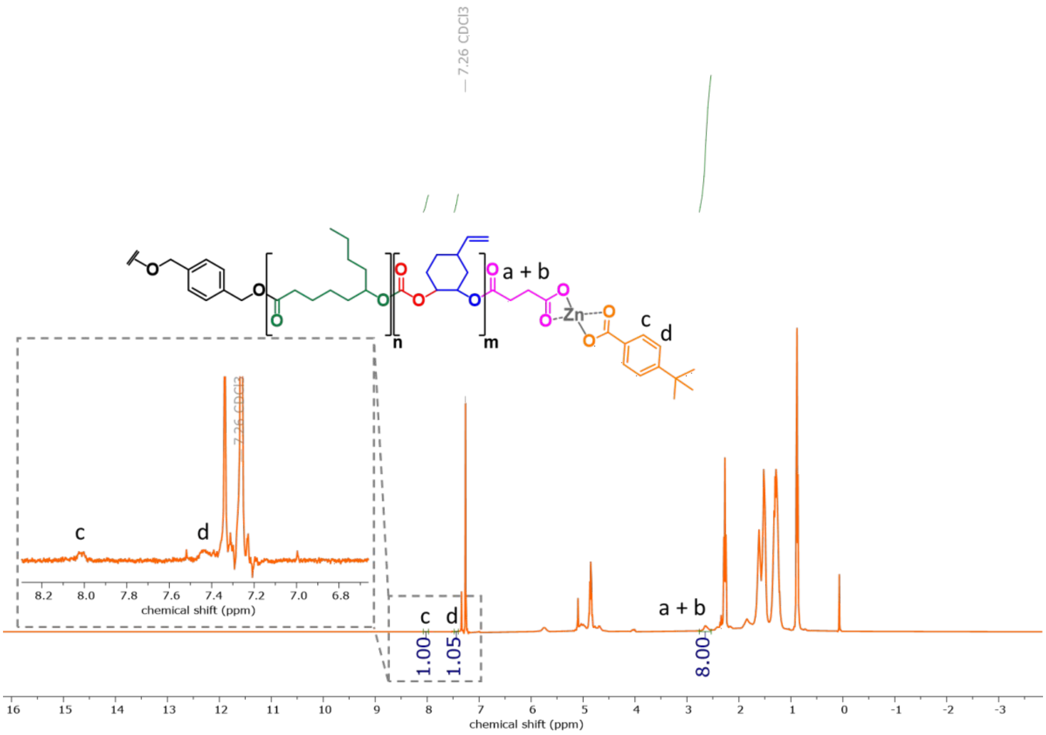

Figure S11: ^{1}H NMR (400 MHz, $CDCl_3$) spectrum of $HP_{Zn,\ 0.50}$.

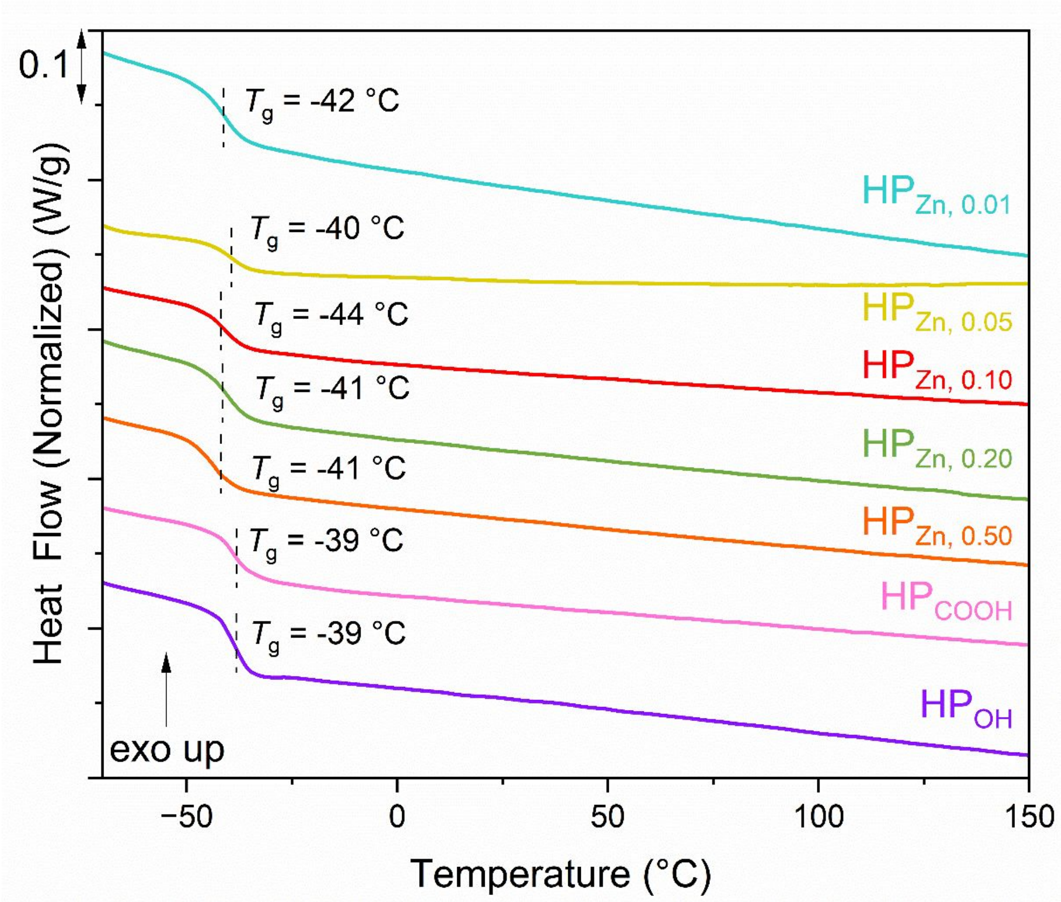

Figure S12: Differential Scanning Calorimetry (DSC) second heating cycle traces of HP_{OH}, HP_{COOH}, $HP_{Zn,\ 0.50}$, $HP_{Zn,\ 0.20}$, $HP_{Zn,\ 0.10}$, $HP_{Zn,\ 0.05}$, $HP_{Zn,\ 0.01}$.

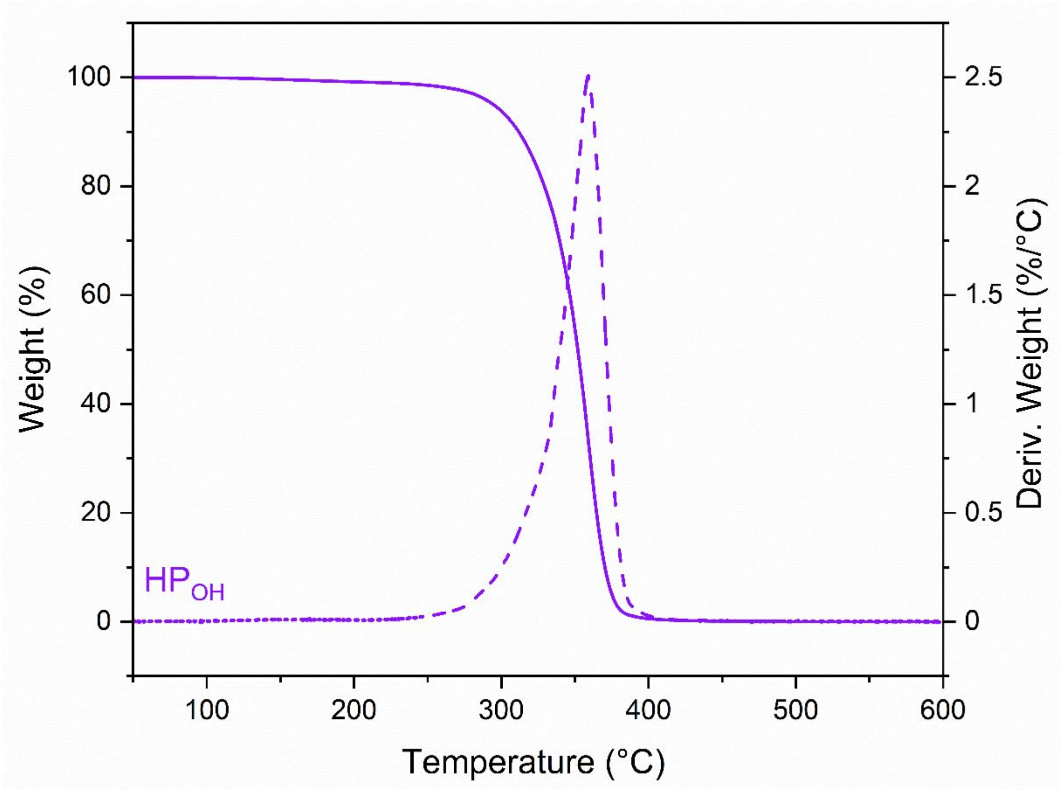

Figure S13: TGA profile for HP$_{OH}$.

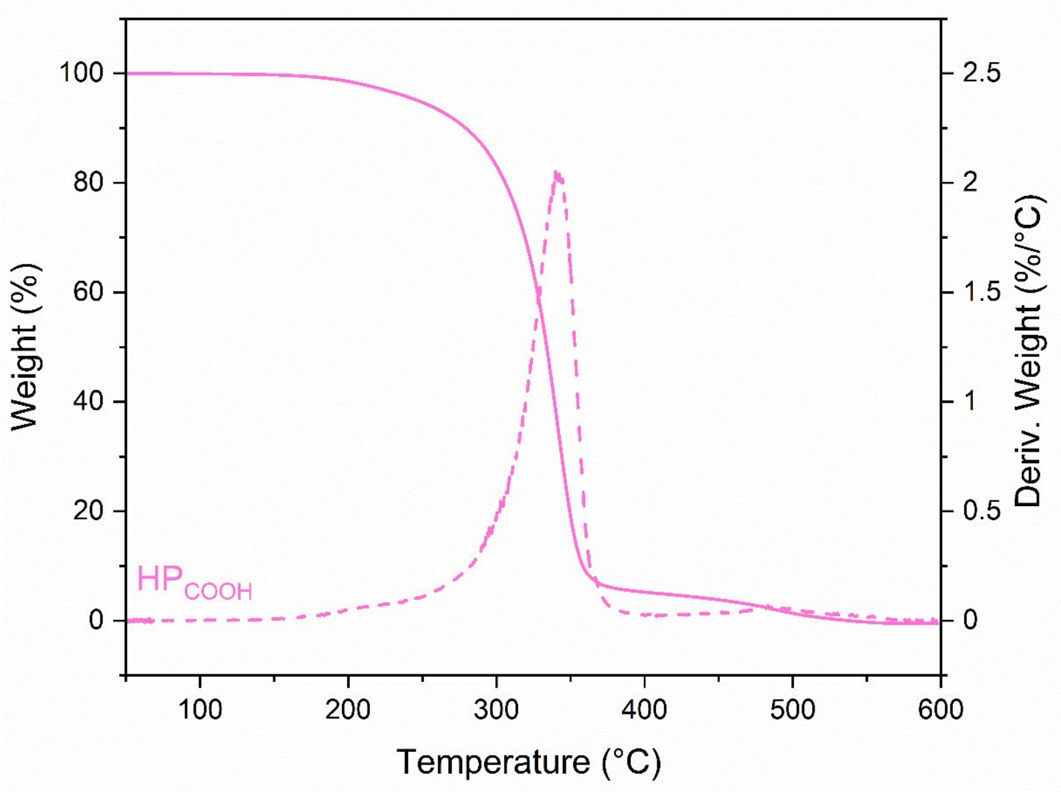

Figure S14: TGA profile for HP$_{COOH}$.

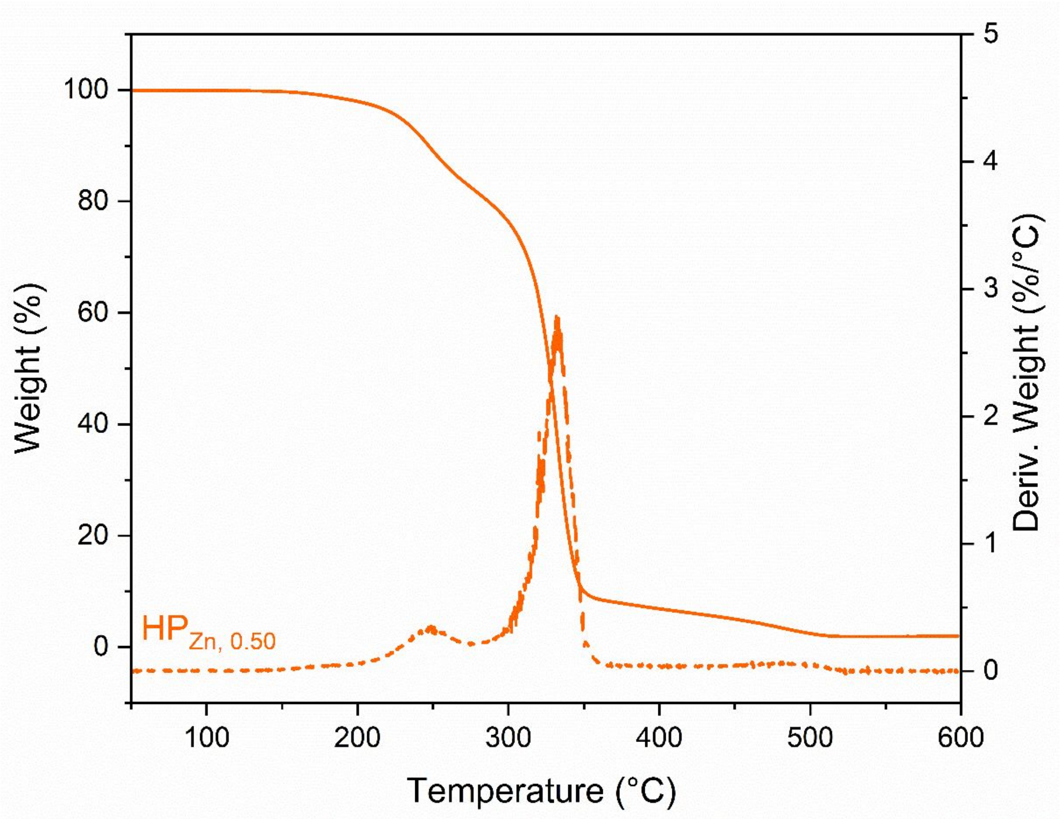

Figure S15: TGA profile for HP$_{Zn, 0.50}$.

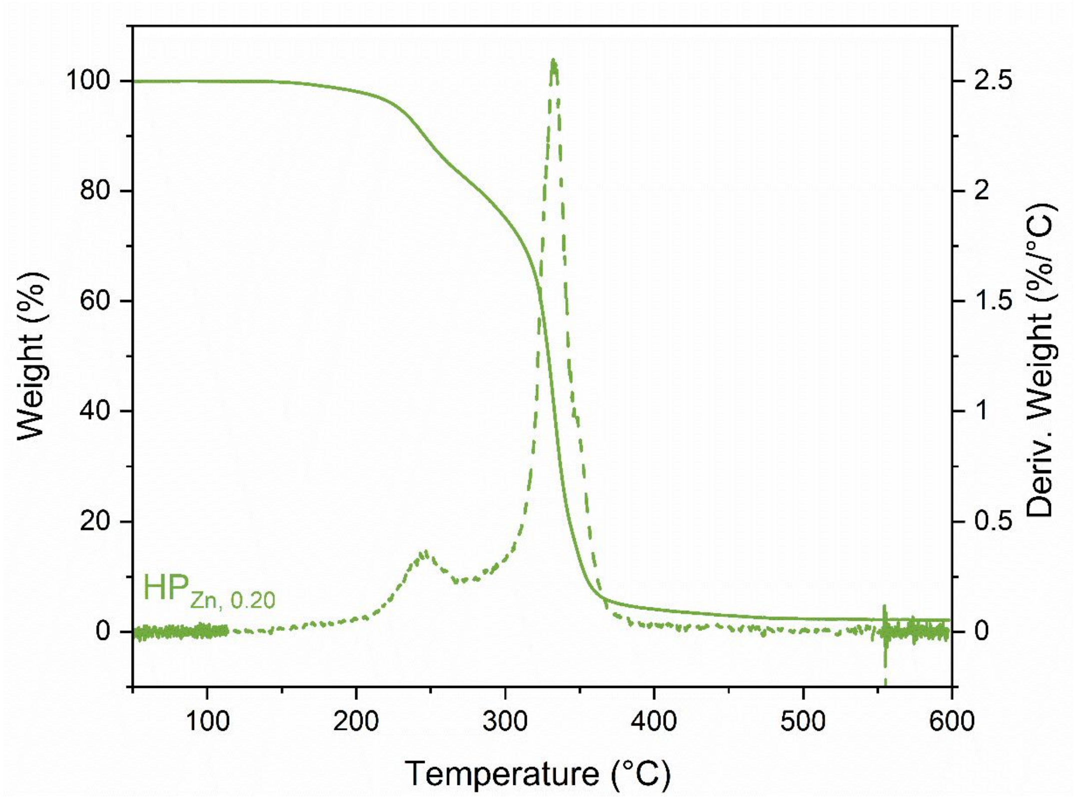

Figure S16: TGA profile for HP$_{Zn, 0.20}$.

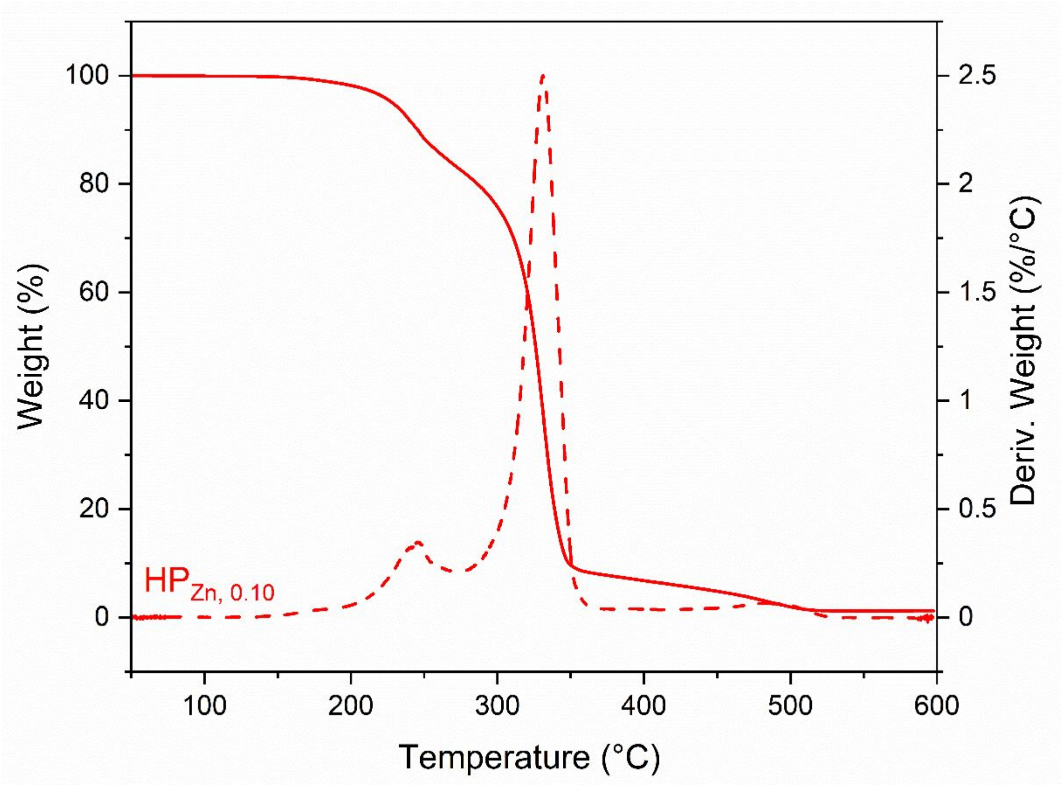

Figure S17: TGA profile for $HP_{Zn,\ 0.10}$.

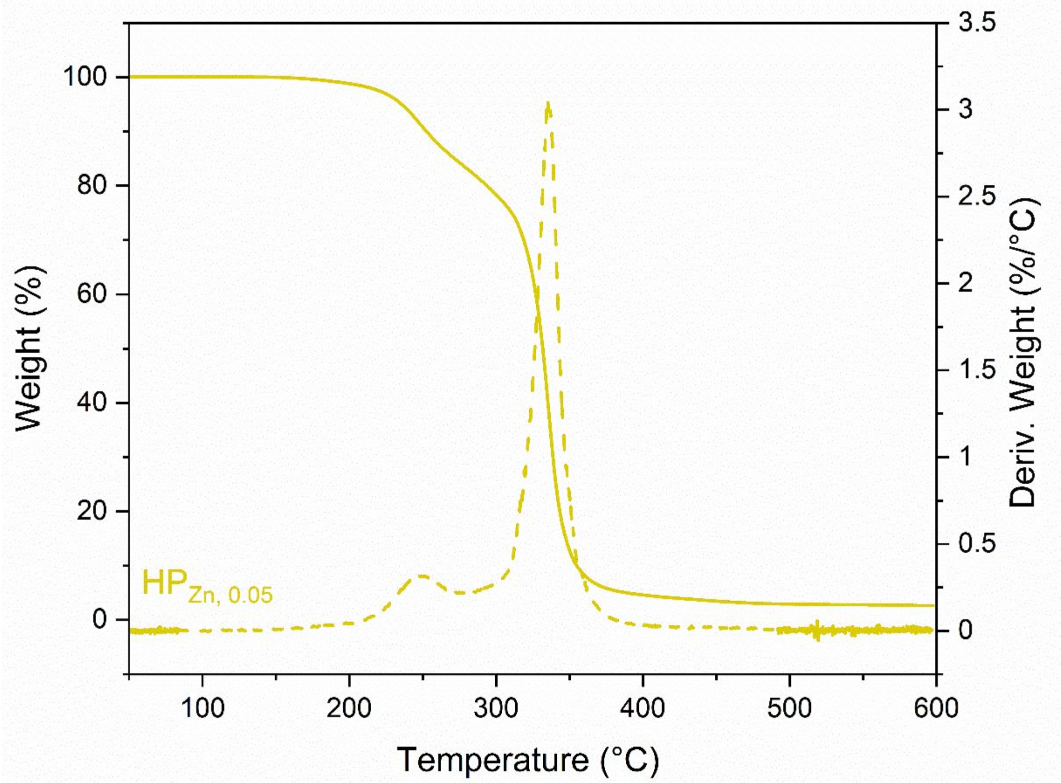

Figure S18: TGA profile for $HP_{Zn,\ 0.05}$.

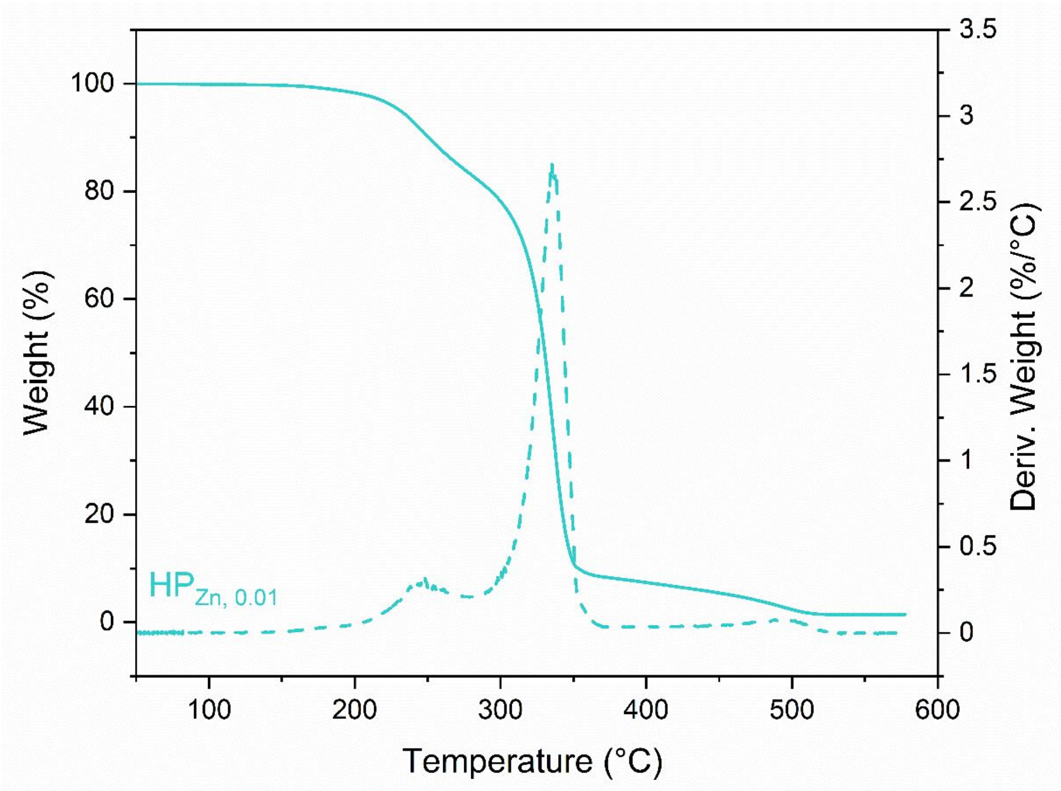

Figure S19: TGA profile for for $HP_{Zn,\ 0.01}$.

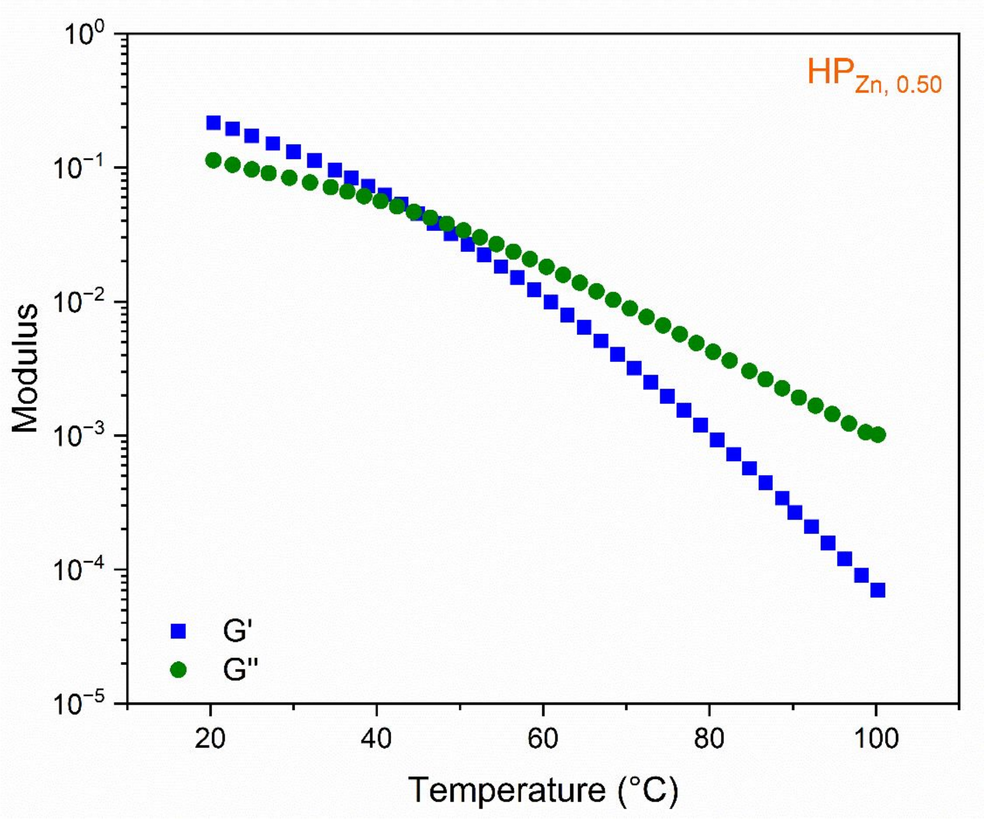

Figure S20: Rheological temperature ramp for $HP_{Zn,\ 0.50}$.

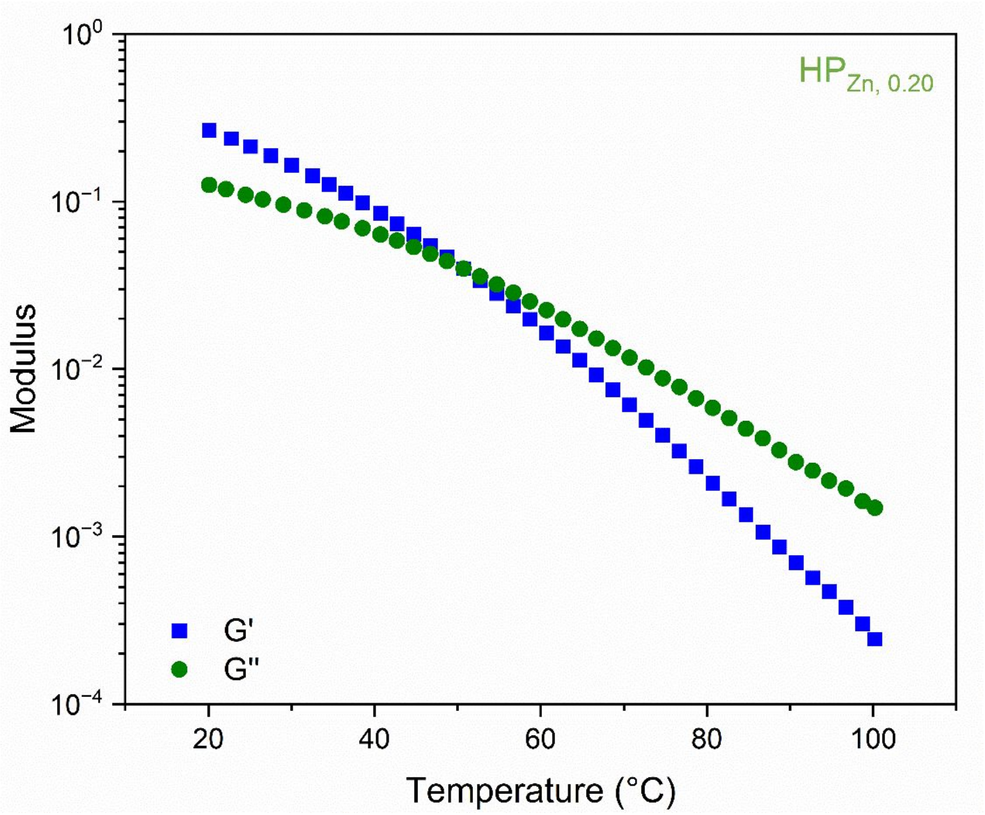

Figure S21: Rheological temperature ramp for $HP_{Zn,\ 0.20}$.

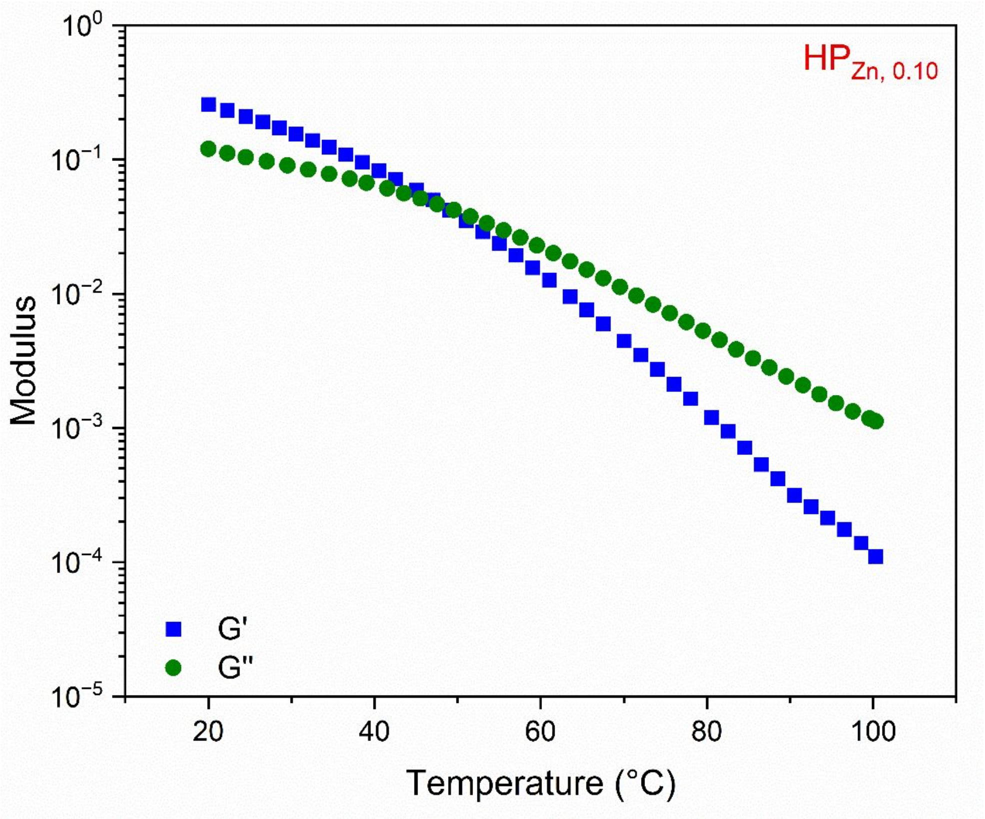

Figure S22: Rheological temperature ramp for $HP_{Zn,\ 0.10}$.

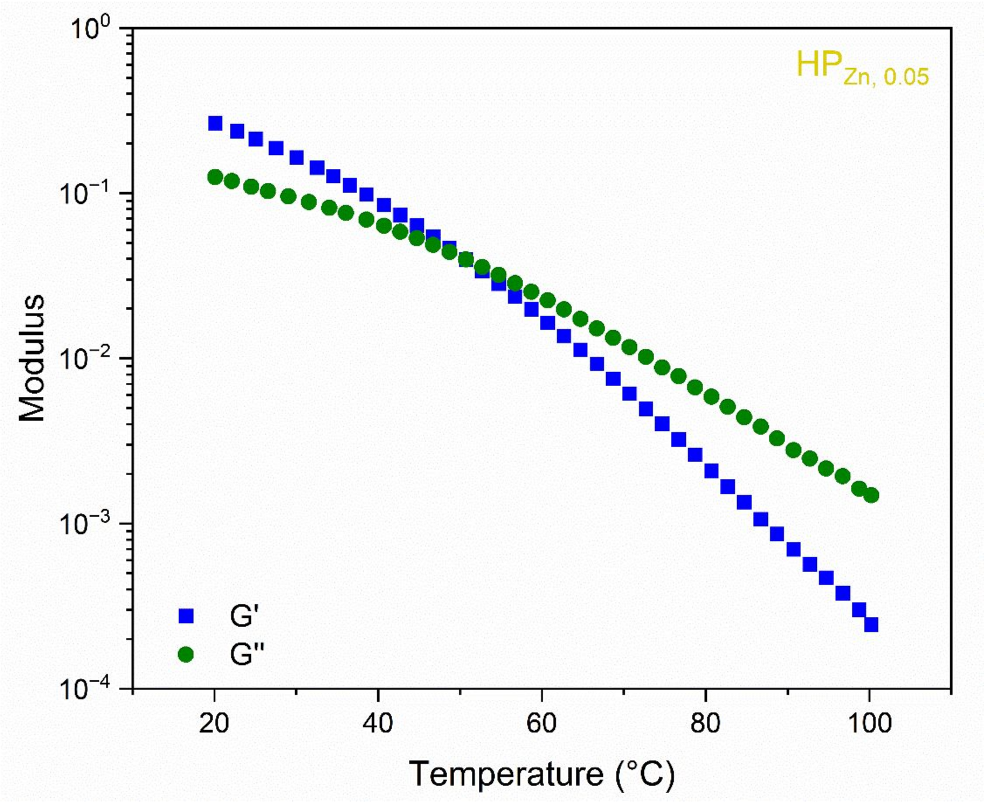

Figure S23: Rheological temperature ramp for HP$_{Zn, 0.05}$.

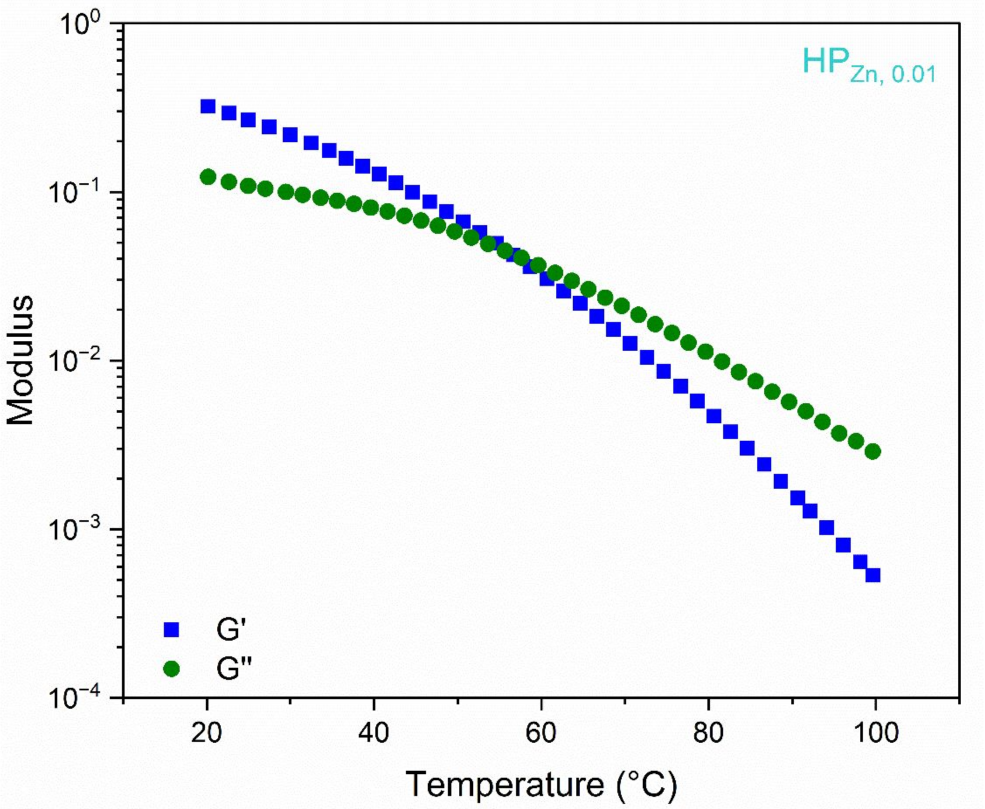

Figure S24: Rheological temperature ramp for HP$_{Zn, 0.01}$.

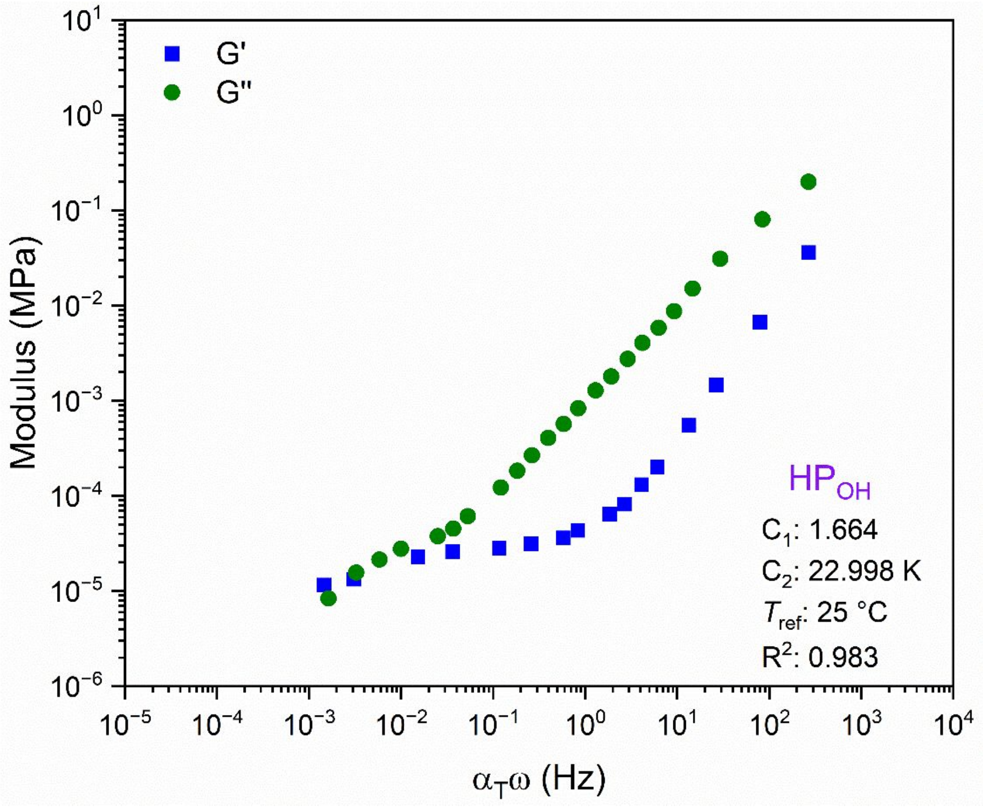

Figure S25: TTS mater curve for HP$_{OH}$.

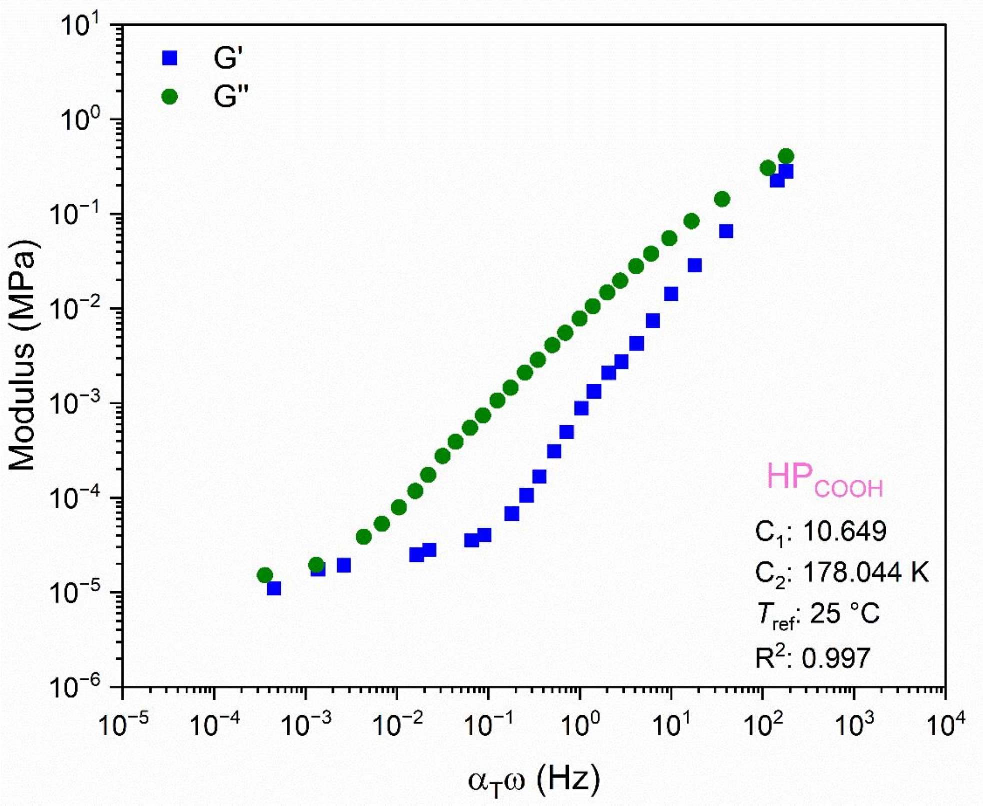

Figure S26: TTS mater curve for HP$_{COOH}$.

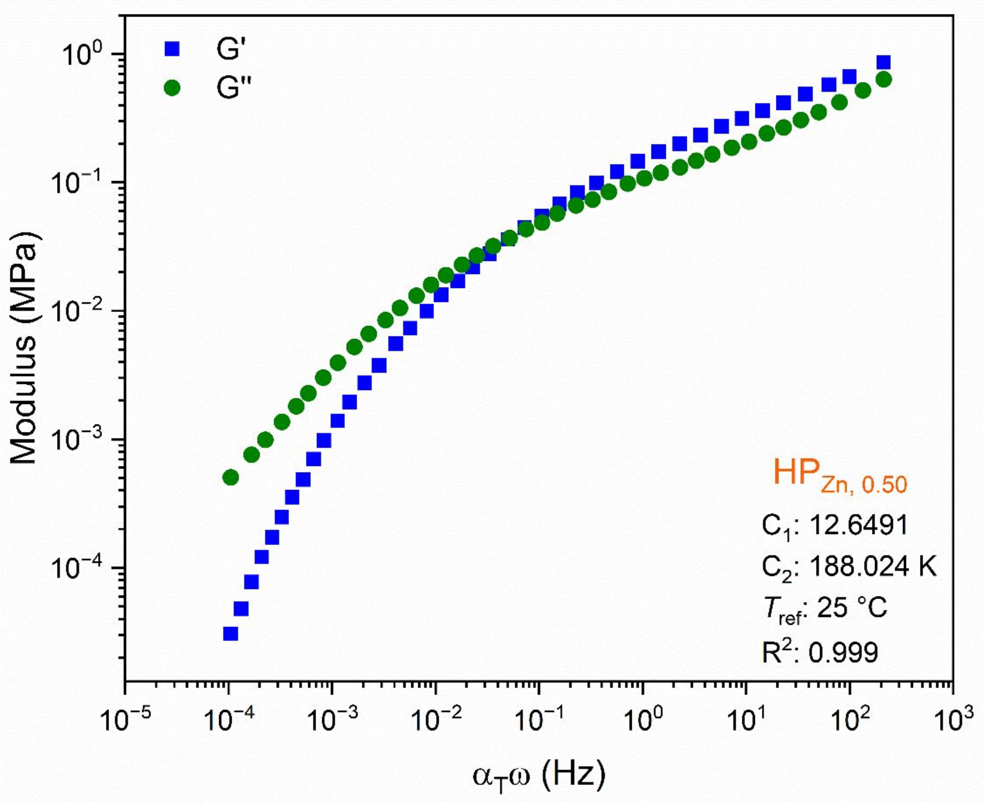

Figure S27: TTS mater curve for $HP_{Zn,\ 0.50}$.

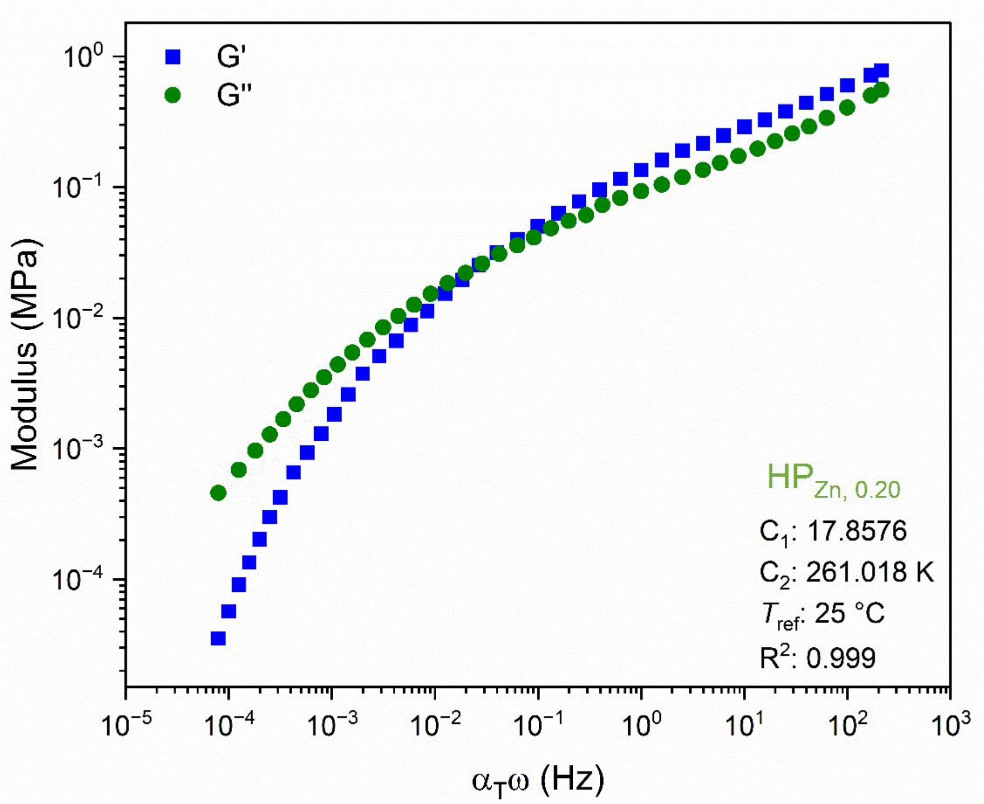

Figure S28: TTS mater curve for $HP_{Zn,\ 0.20}$.

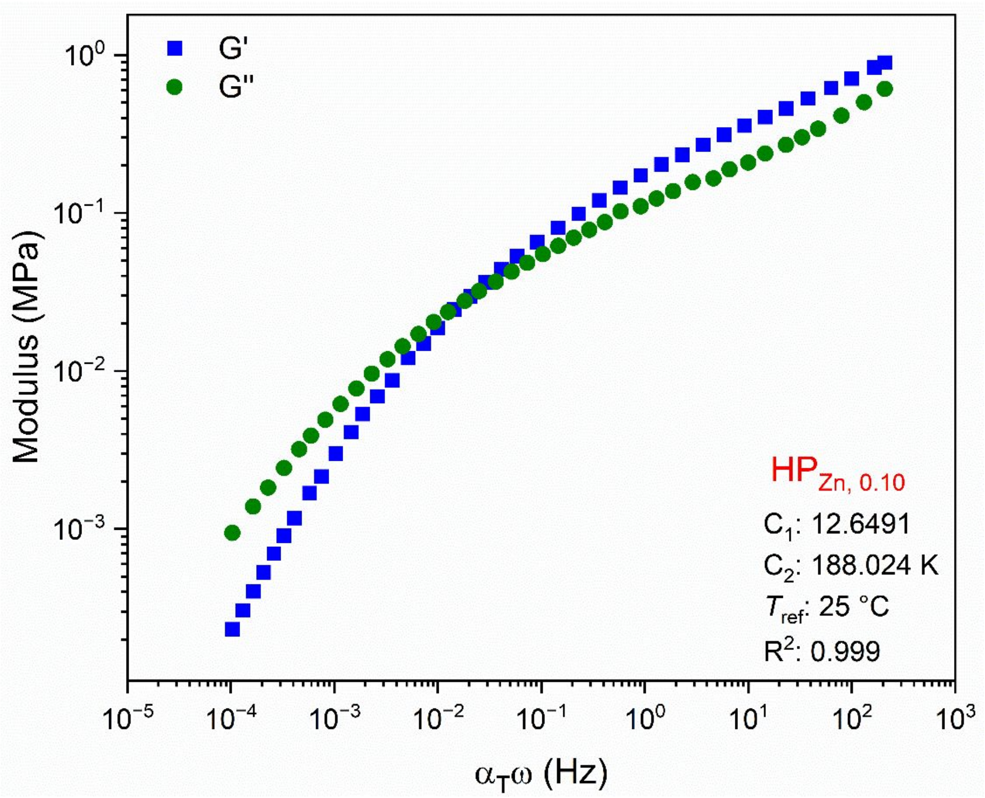

Figure S29: TTS mater curve for $HP_{Zn,\ 0.10}$.

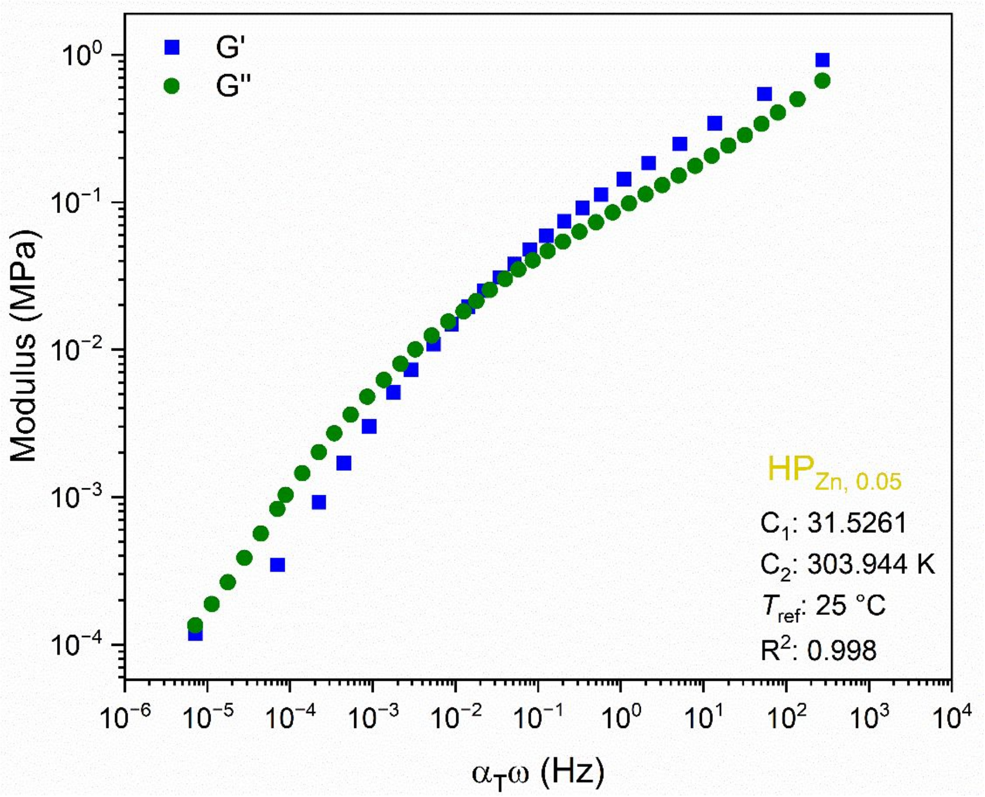

Figure S30: TTS mater curve for $HP_{Zn,\ 0.05}$.

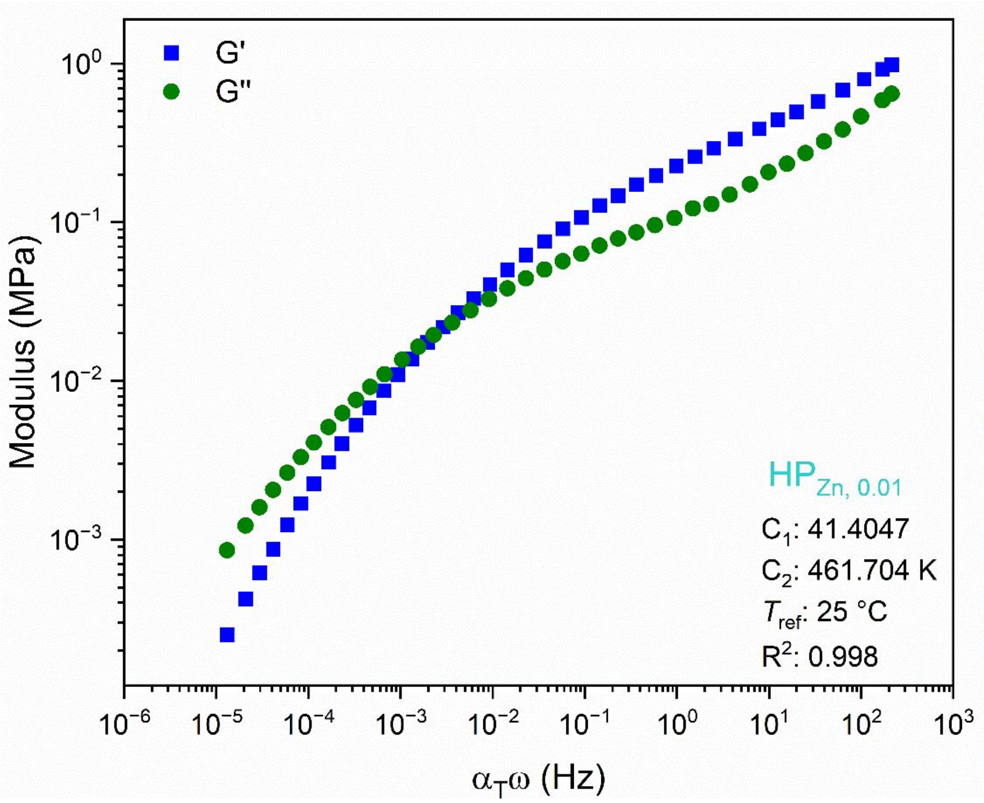

Figure S31: TTS mater curve for HP$_{Zn, 0.01}$.

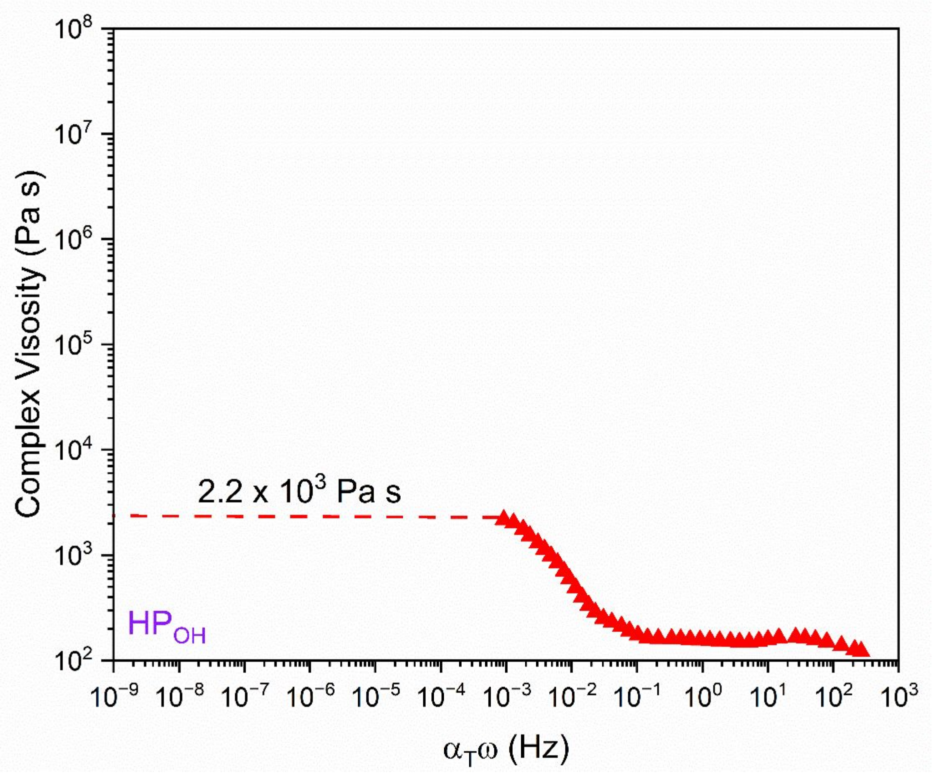

Figure S32: Plot of shear viscosity vs frequency (Hz) obtained from TTS master curves and projection of the plateau viscosity to zero-shear for HP$_{OH}$. Note: The complex viscosity does not fully plateau in the time–temperature superposition, so the reported zero-shear viscosity is likely a slight underestimation.

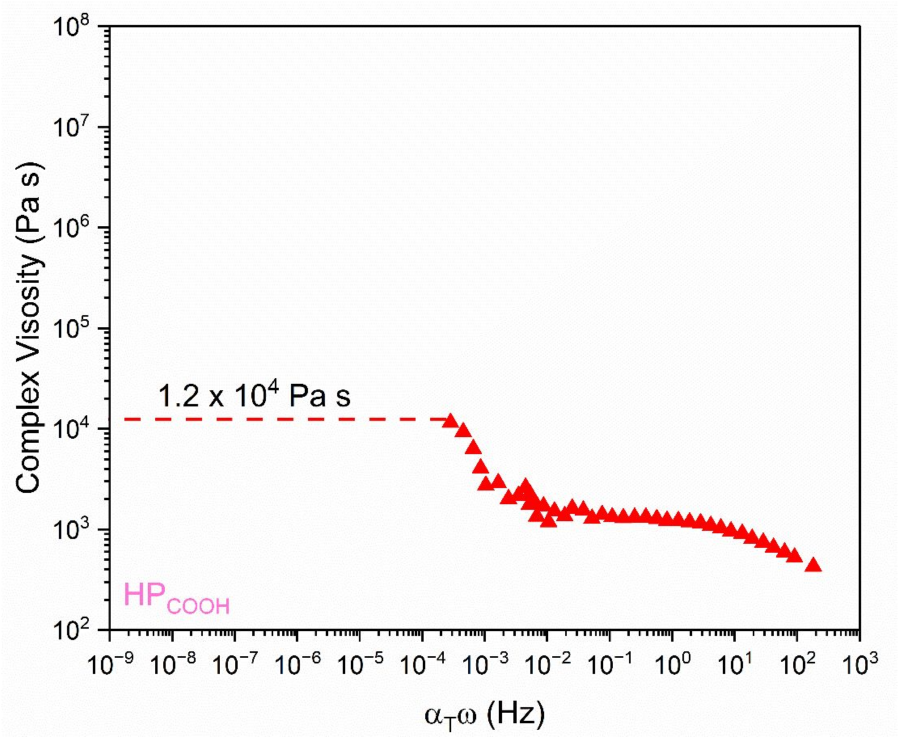

Figure S33: Plot of shear viscosity vs frequency (Hz) obtained from TTS master curves and projection of the plateau viscosity to zero-shear for HP_{COOH}. Note: The complex viscosity does not fully plateau in the time–temperature superposition, so the reported zero-shear viscosity is likely a slight underestimation.

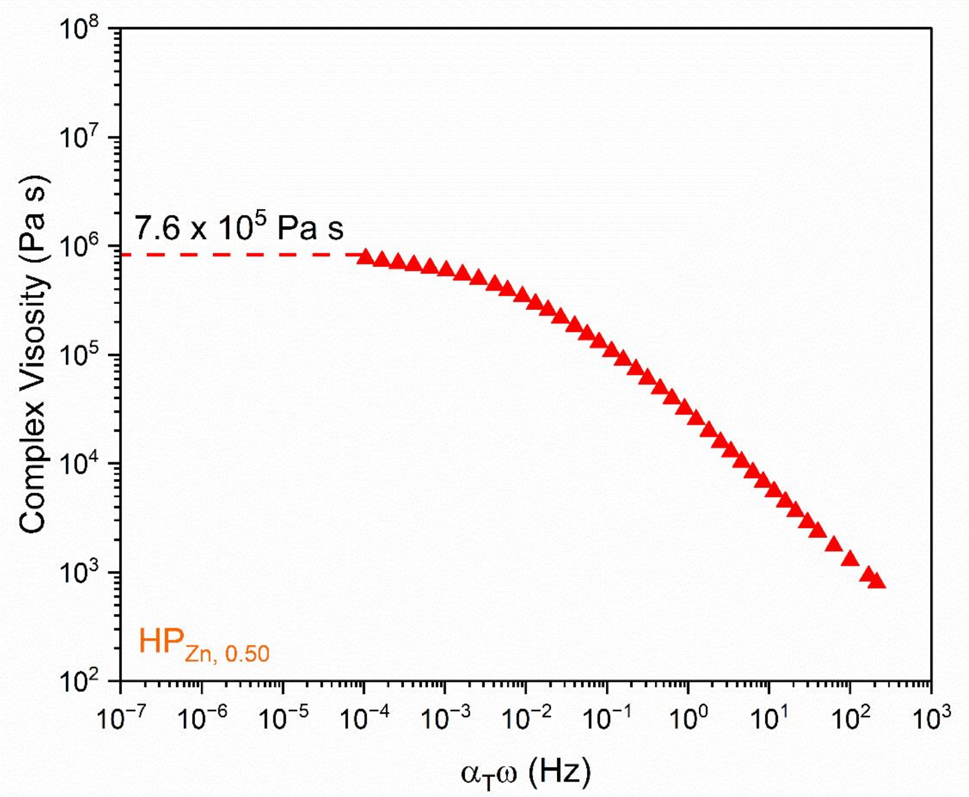

Figure S34: Plot of shear viscosity vs frequency (Hz) obtained from TTS master curves and projection of the plateau viscosity to zero-shear for $HP_{Zn,\ 0.50}$.

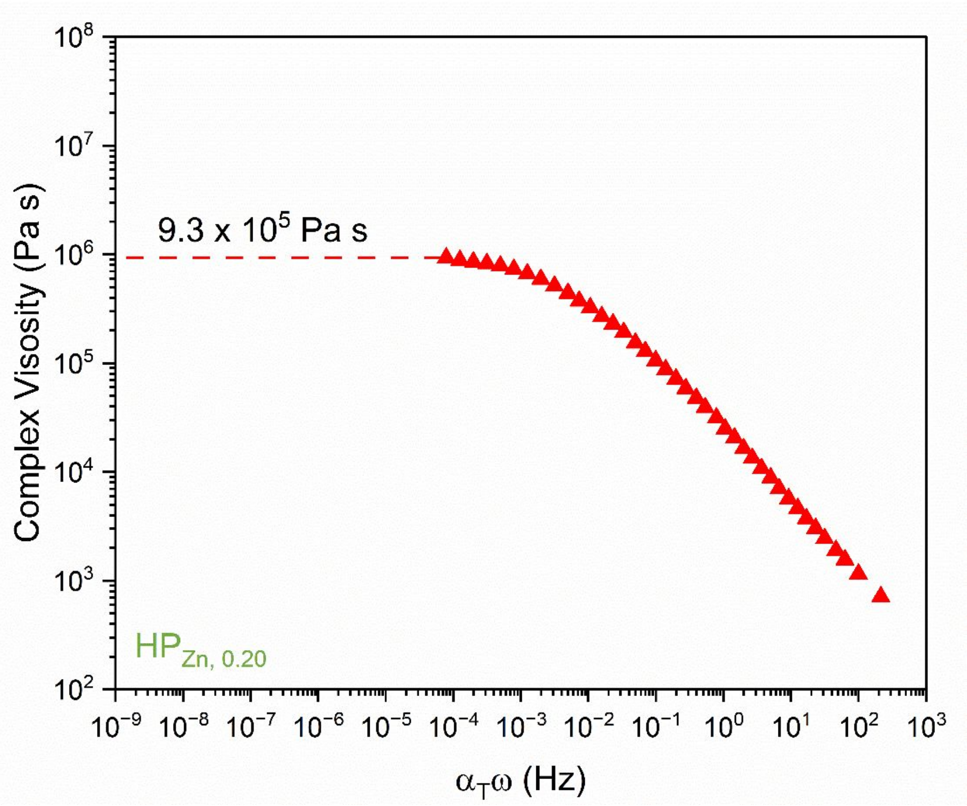

Figure S35: Plot of shear viscosity vs frequency (Hz) obtained from TTS master curves and projection of the plateau viscosity to zero-shear for $HP_{Zn,\ 0.20}$.

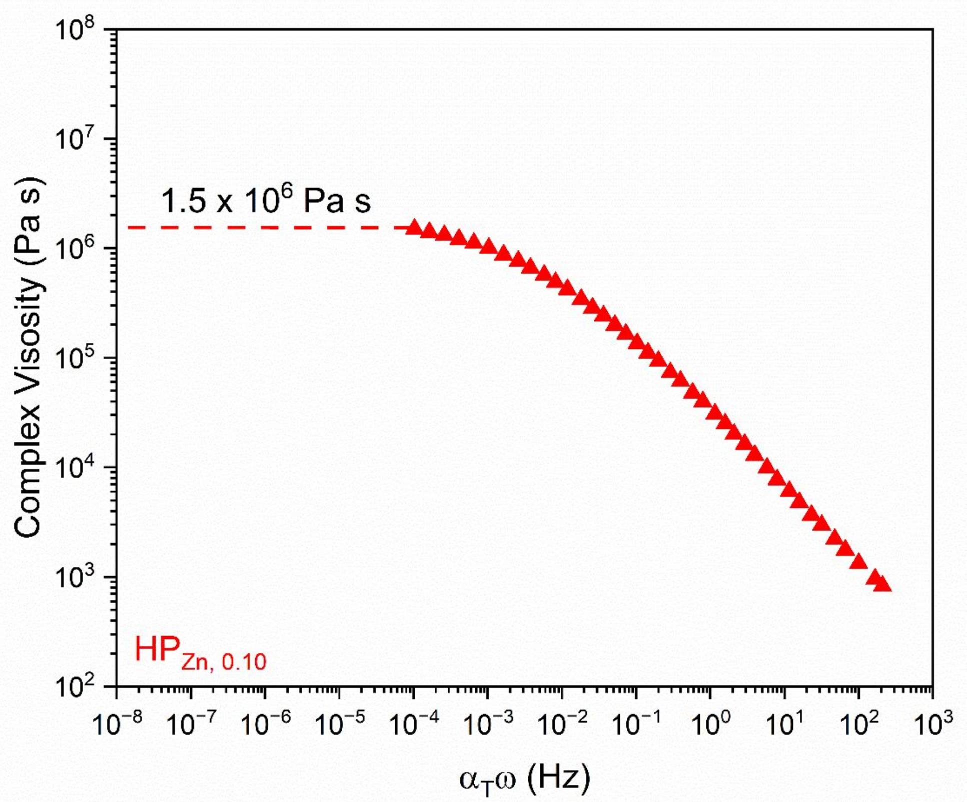

Figure S36: Plot of shear viscosity vs frequency (Hz) obtained from TTS master curves and projection of the plateau viscosity to zero-shear for $HP_{Zn,\ 0.10}$.

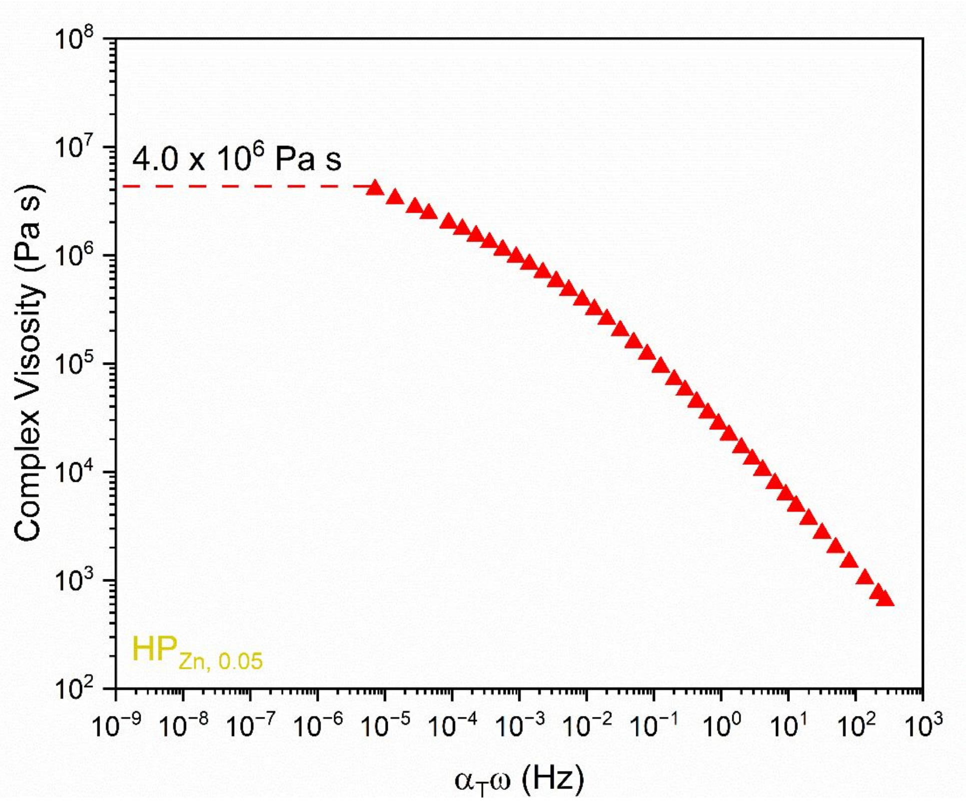

Figure S37: Plot of shear viscosity vs frequency (Hz) obtained from TTS master curves and projection of the plateau viscosity to zero-shear for $HP_{Zn,\ 0.05}$.

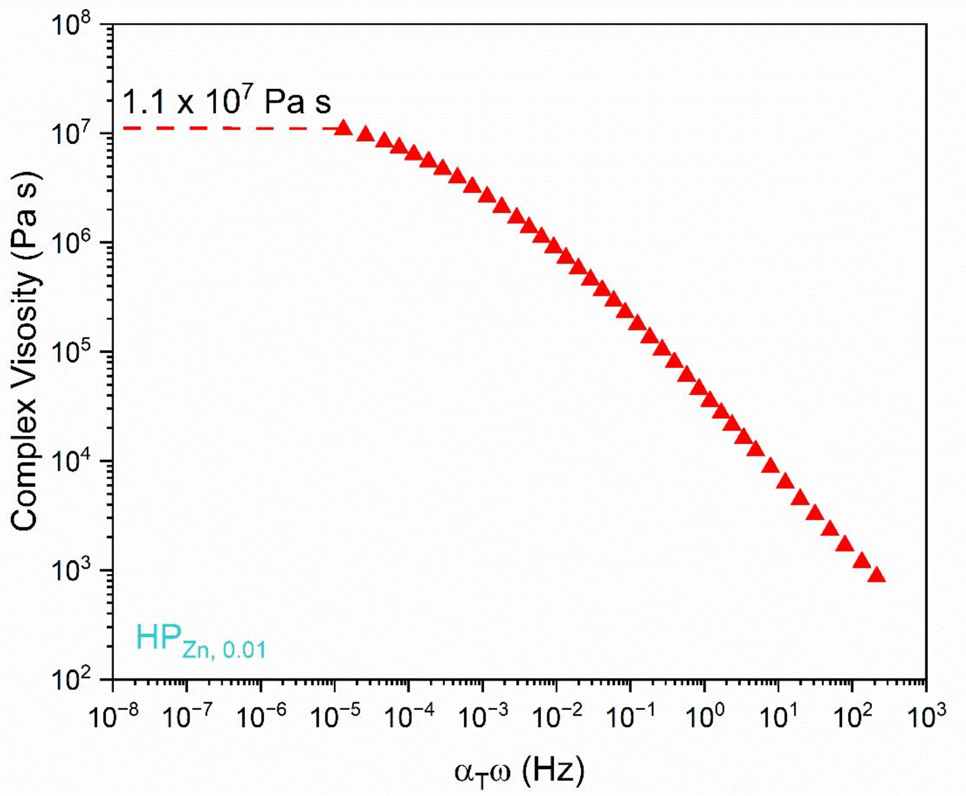

Figure S38: Plot of shear viscosity vs frequency (Hz) obtained from TTS master curves and projection of the plateau viscosity to zero-shear for $HP_{Zn,\ 0.01}$.

References

(1) Spyros, A.; Argyropoulos, D. S.; Marchessault, R. H. A study of poly(hydroxyalkanoate)s by quantitative P-31 NMR spectroscopy: Molecular weight and chain cleavage. *Macromolecules* **1997**, *30* (2), 327–329. DOI: DOI 10.1021/ma9601979.
(2) Deacy, A. C.; Kilpatrick, A. F. R.; Regoutz, A.; Williams, C. K. Understanding metal synergy in heterodinuclear catalysts for the copolymerization of CO and epoxides. *Nat Chem* **2020**, *12* (4), 372–380. DOI: 10.1038/s41557-020-0450-3.
(3) Poon, K. C.; Segal, M.; Bahnick, A. J.; Chan, Y. M.; Gao, C.; Becker, M. L.; Williams, C. K. Digital Light Processing to Afford High Resolution and Degradable CO2-Derived Copolymer Elastomers. *Angew. Chem. Int. Ed.* **2024**, *63* (33), e202407794. DOI: 10.1002/anie.202407794.
